# Ligands modification strategies for mononuclear water splitting catalysts

**DOI:** 10.3389/fchem.2022.996383

**Published:** 2022-09-27

**Authors:** Lei Wang, Lijuan Wang

**Affiliations:** School of Materials and Chemistry, University of Shanghai for Science and Technology, Shanghai, China

**Keywords:** molecular catalysts, modification strategies, ligands design, mononuclear complexes, water splitting

## Abstract

Artificial photosynthesis (AP) has been proved to be a promising way of alleviating global climate change and energy crisis. Among various materials for AP, molecular complexes play an important role due to their favorable efficiency, stability, and activity. As a result of its importance, the topic has been extensively reviewed, however, most of them paid attention to the designs and preparations of complexes and their water splitting mechanisms. In fact, ligands design and preparation also play an important role in metal complexes’ properties and catalysis performance. In this review, we focus on the ligands that are suitable for designing mononuclear catalysts for water splitting, providing a coherent discussion at the strategic level because of the availability of various activity studies for the selected complexes. Two main designing strategies for ligands in molecular catalysts, substituents modification and backbone construction, are discussed in detail in terms of their potentials for water splitting catalysts.

## 1 Introduction

To address the problems of climate change and energy crisis, solar energy technologies have been developing and applying for decades thanks to the abundant, renewable energy sources of Sun light. Although photovoltaic technologies can convert solar energy into electrical energy, the energy conversion and utilization are highly dependent on the weather and time of a day. The intermittent and diffuse nature of solar energy and the need for taking full advantages of Sun light promote the development of more efficient storage technologies for solar energy (Akbari et al., 2019; Liu et al., 2019; Palacios et al., 2020).

Inspiring from the natural photosynthesis process, during which one oxygen, four protons and four electrons are liberated in water oxidation phase then the protons and electrons contributed to carbon dioxide fixation in the photosystem II (PSII), artificial photosynthesis has been extensively studied and is considered as an attractive technology to produce green and sustainable energy (Whang and Apaydin, 2018; Ye et al., 2018; Dogutan and Nocera, 2019; Zhang and Reisner, 2020). In artificial photosynthesis, water splitting including oxygen-evolving reaction (OER), Eq. 1, and hydrogen-evolving reaction (HER), Eq. 2, is attractive for the solar energy utilization and storage, where OER, due to the thermodynamical and kinetical barriers, is the bottleneck of this process.

$${\text{2H}}_{\text{2}}\text{O}\to {\text{O}}_{\text{2}}{\text{ + 4H}}^{\text{+}}{\text{+4e}}^{\text{-}}\left(\text{E = 1}\text{.23 V vs NHE}\right)$$ (1)

$${\text{2H}}^{\text{+}}{\text{+2e}}^{\text{-}}\to {\text{H}}_{\text{2}}\left(\text{E = 0 V vs NHE}\right)$$ (2)

**FIGURE 1:**
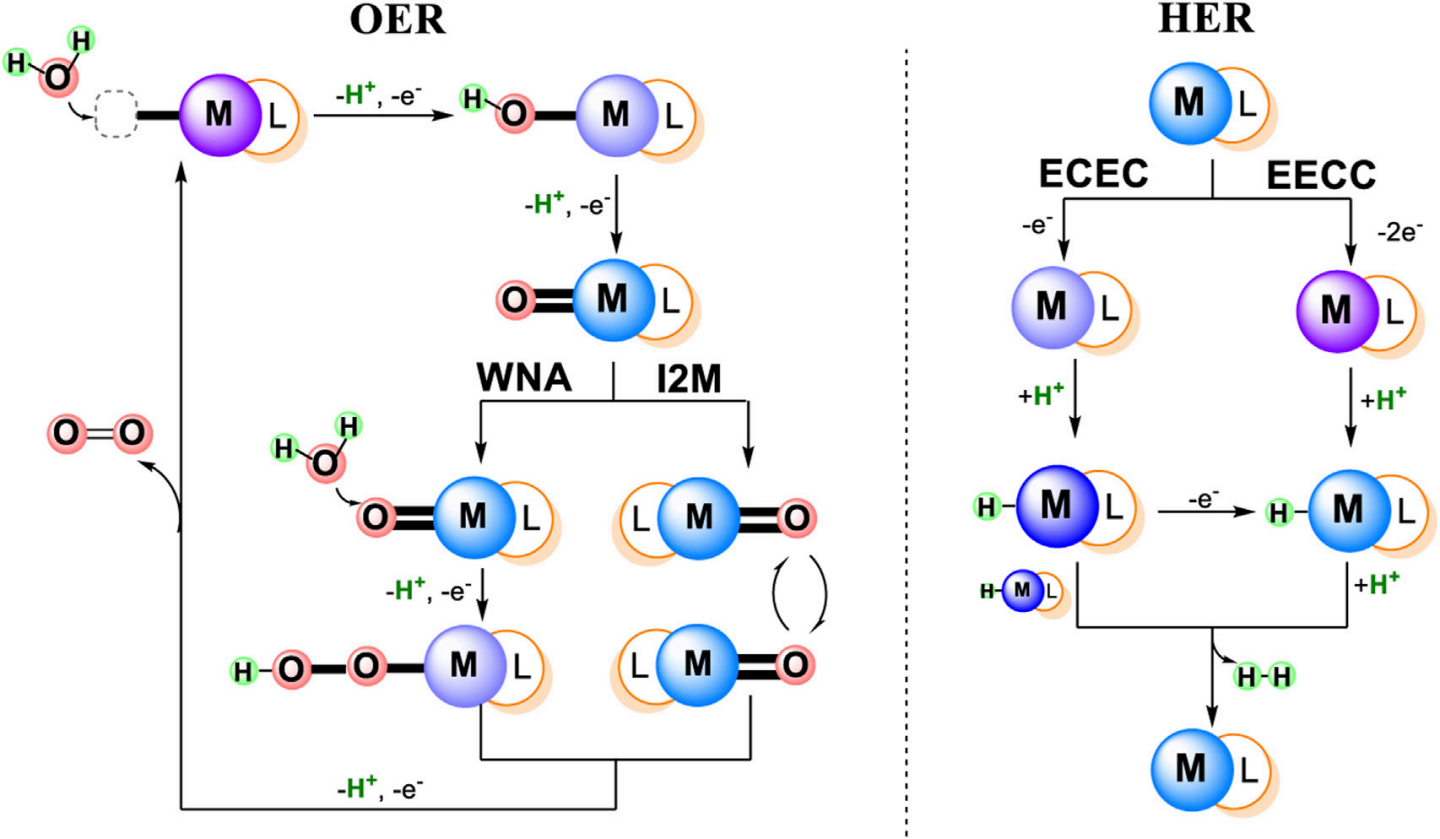
Schematic representation of general mechanisms for Water Oxidation and Water Reduction catalyzed by metal complexes.

There are two major mechanistic classifications for each water splitting process: 1) OER reaction: I2M, WNA, and 2) HER reaction: ECEC, EECC, as shown in Figure 1 (Blakemore et al., 2015; Shaffer et al., 2017; Wang et al., 2019). The general two types of OER mechanisms both involve O-O bond formation, where an O-O radical coupling interaction of two metallo-oxy radicals are involved in the coupling (I2M mechanism) or a water molecular attack on an electrophilic metal-oxo or metal-oxyl (WNA mechanism) (Shaffer et al., 2017). The two types of HER mechanisms are distinguished by the initial reduction: 1) one electron and two H-M^(n−1)+^ react with each other to generate H_2_ (ECEC mechanism), 2) two electrons and the resultant M^(n−2)+^ can be protonated to H-M^n+^, which then can react with an external proton to yield H_2_ (EECC mechanism) (Wang et al., 2019).

Among catalysts for water splitting, molecular metal complexes have been paid tremendous attention due to the following advantages: 1) designable steric configuration and electronic structure; 2) tunable intrinsic activity; 3) clear catalytic mechanisms; 4) high selectivity of products 5) high atomic economy; 6) compatible with the development of various spectroscopic instruments. Over the last few decades, many efficient molecular water oxidation catalysts (WOCs) and water reduction catalysts (WRCs) were developed, such as the ruthenium catalysts (Concepcion et al., 2009b), iridium catalysts (Moore et al., 2011), manganese catalysts (Najafpour and Allakhverdiev, 2012), cobalt catalysts (Eckenhoff et al., 2013), platinum catalysts (Wang et al., 2015), iron catalysts (Mehrabani et al., 2020), copper catalysts (Liu et al., 2018), and nickel catalysts (Shaffer et al., 2017; Wang et al., 2019) etc. Thereby, many reviews are available in the literature, comparing various kinds of molecular catalysts comprehensively and summarizing catalytic mechanisms for water splitting (Meyer et al., 2017; Stolarczyk et al., 2018; Zhang and Sun, 2019a).

Several procedures are involved in developing molecular catalysts for water splitting: 1) ligand design, synthesis, and characterization; 2) metal complexes synthesis and performance characterization; 3) catalytic mechanism studies. In fact, the flourish of various ligands designed for WOCs and WRCs, as well as active metal site, establish the foundation of molecular catalysts performance. The combination of different metals and ligands will create thousands of molecular catalysts. Therefore, the modification strategies of ligands are recognized as one of the challenges to improve the intrinsic catalytic activity and stability of water splitting catalysts (WSCs).

**FIGURE 2:**
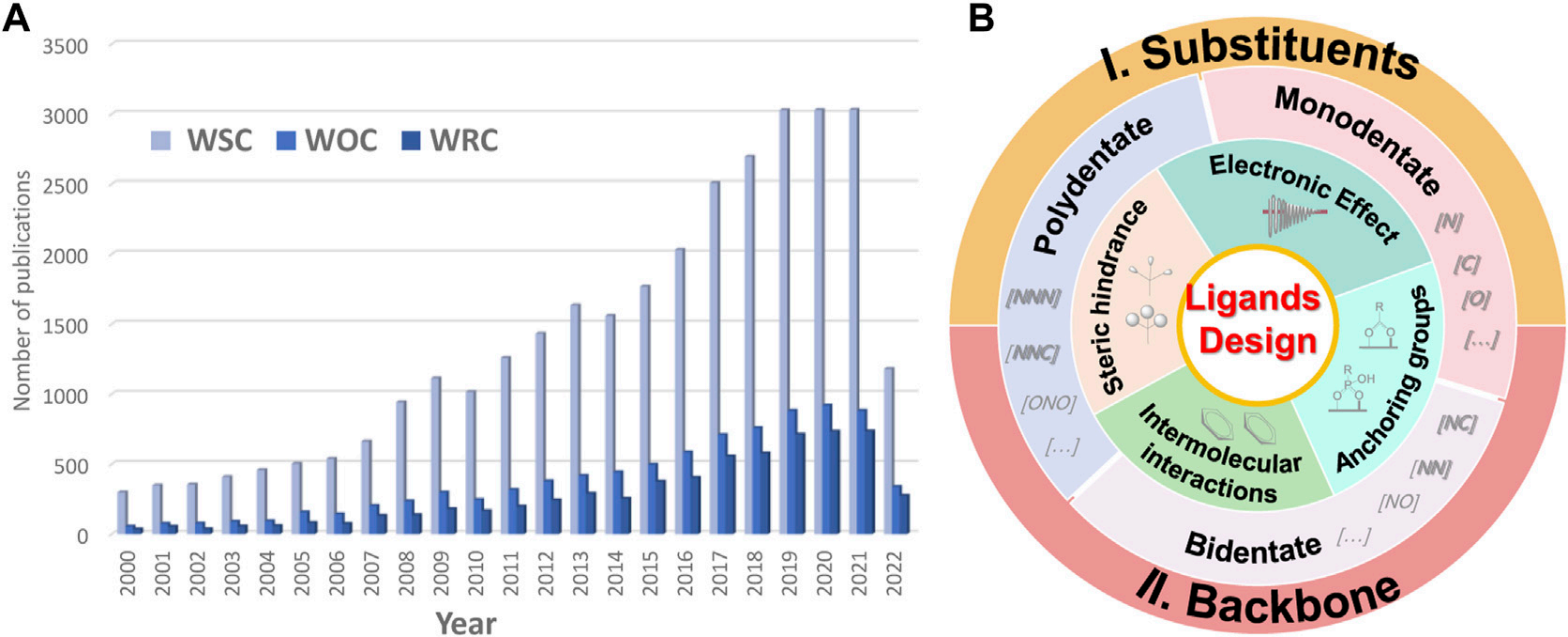
**(A)** Number of published papers related to molecular catalysts for water splitting from 2000 to 2022 (data obtained on 30 July 2022, from a search performed in the Web of Science for “molecular water splitting*“, “molecular water oxidation*“, and “molecular water reduction*” topics). **(B)** Designing strategies of ligands for molecular water splitting catalysts.

In this review, we emphasize the modification strategies of ligands and their effect on the properties and performance of WSCs. We address here the two major designing strategies of ligands for mononuclear water splitting outlined in Figure 2B, including 1) substituents modification, and 2) backbone construction. We consider four strategies for substituents modification, including electronic effect, intermolecular interactions, steric hindrance, and anchoring groups, with providing corresponding examples for each of them. The backbone construction refers to the parent configurations that are organized by coordination number, including monodentate, bidentate, and polydentate ligands. In the end, we summarize the ligands’ designing strategies and highlight their prospects in future research of molecular complexes for artificial photosynthesis.

## 2 Substituent-modification strategies of ligands

The large variety of organic substituents as well as the straightforward synthesis of both ligands and metal complexes open a large new window for the design of ligand-based WSCs. Though the same type of ligands may have different effect on WSCs’ performance depending on the mechanism of catalysts and the central active metal site, the major substituents modification strategies can be briefly summarized as: electronic effect, intramolecular interactions, steric hindrance, and anchoring groups.

### 2.1 Electronic effect

Over the last decade, the exploration of electronic effect on the properties of metal complexes has been dramatically increased owning to its easy-monitored nature by various methods (Allen and Cook, 1963; Lanznaster et al., 2006; Kieltsch et al., 2010; Feng et al., 2014; Chen et al., 2015; Bellows et al., 2016; Matheu et al., 2019a; Meza-Chincha et al., 2020). It is worth to note that the electronic effect can usually be reflected by the Hammette parameter σ (σ_m_ or σ_p_, depending on the position of substituent), which increases with the increasing of electron-withdrawing ability. Hansch et al. (1991) summarized σ values of various substituents, reporting that electron-withdrawing groups such as -CF_3_ and -Br possess positive values while the electron-donating groups such as -NH_2_ and -OEt have negative values, and σ of hydrogen (H) equals to zero. As a result, a plenty of works studied the relationships between σ and redox potential, λ, or reactivity, etc (Clark et al., 2018; Zhang et al., 2018; Suresh et al., 2022).

**FIGURE 3:**
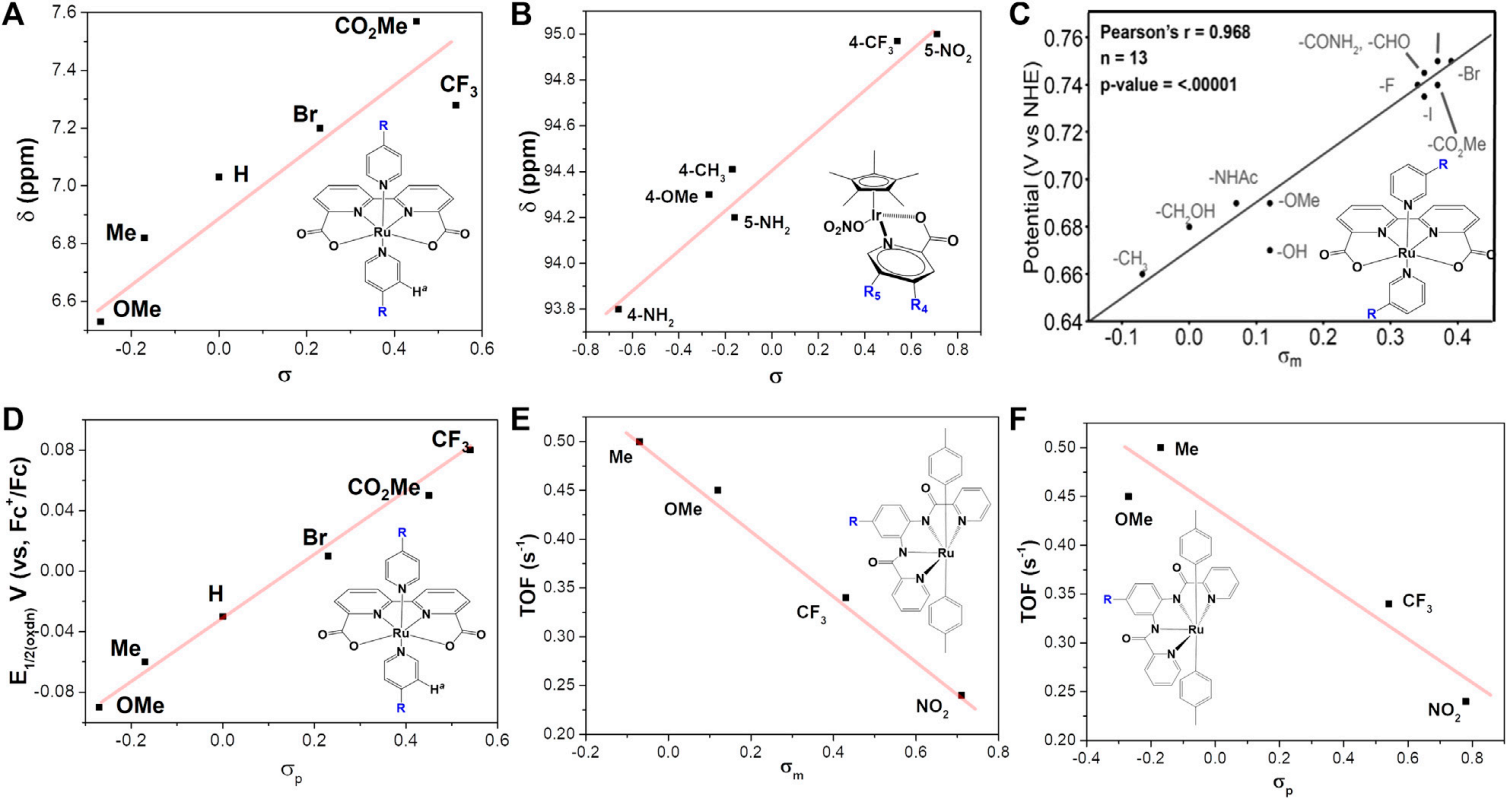
Plot of σ-Hammett parameter versus **(A)** ^1^H NMR chemical shift of ortho-proton on the pyridine ligands, data from Sato et al. (2015), **(B)** ^13^C NMR chemical shift of quaternary carbon atom on the Cp, data from Rodriguez et al. (2021), **(C)** Redox couples Ru^III^/Ru^II^, adapt with permission from Angew. Chem. 2021,133,14625–14632. Copyright 2021 Angewandte Chemie published by Wiley-VCHGmbH, **(D)** The oxidation potential of [Ru (bda)-(pyR)_2_], Sato et al. (2015), **(E,F)** Turnover frequency (TOF) for complex **3**, data from Abdel-Magied et al. (2017).

**FIGURE 4:**
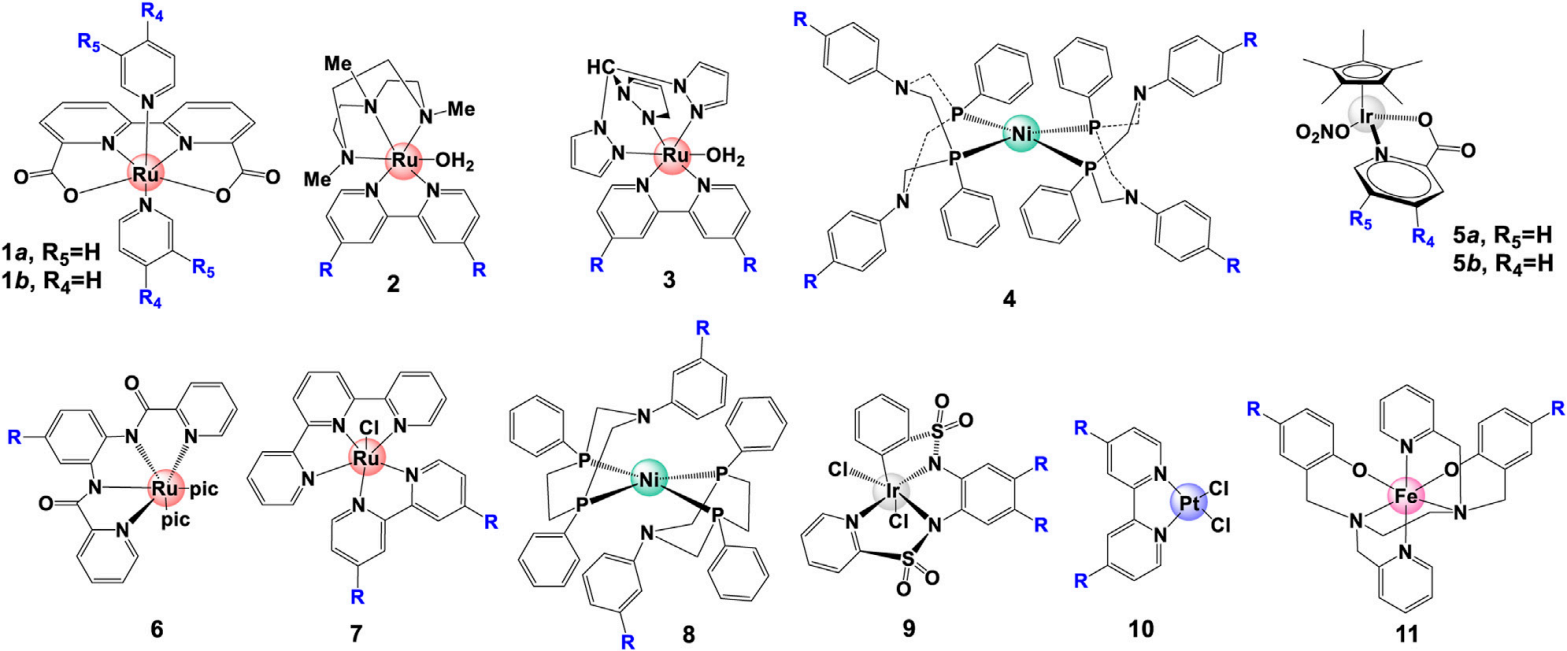
Selected water splitting catalysts with electron-withdrawing/-donating substituents.

The electronic changes have notable effects on the electron density over metal center, resulting in changes of NMR spectra and electrochemical properties. NMR analysis from previous studies (Figures 3A,B) demonstrated that an electron-donating group causes significant up-shifting of protons in ligand(s), and an electron-withdrawing group has the opposite effect, i.e., lower the field chemical shifts of protons in NMR spectra (An et al., 2012; Duan et al., 2013; Sato et al., 2015). In general, electron-withdrawing groups decrease electronic density and stabilize the metal’s lower oxidation state, leading to more positive redox potentials as well as the overpotentials and less back-bonding into coordinated ligands. On the contrary, electron-donating groups can increase the stability of the higher oxidation state *via* increasing the electronic density over the metal center, resulting in more back-bonding interactions with coordinated ligands (Yoshida et al., 2010; Garrido-Barros et al., 2015). Typical examples of this case are WO catalysts [Ru (bda)L_2_] (complex **1a**) (Duan et al., 2013; Sato et al., 2015), HER catalyst [NiP_2_^Ph^N_2_^C6H4X^]_2_ (complex **4**) (Kilgore et al., 2011a) and Pt (bpy-R_2_)Cl_2_ (complex **10**) (Mcinnes et al., 1999), shown in Figure 4. The electrochemical investigation illustrated that the overpotential is drastically reduced as the electron-donating ability increases, and the redox potentials become more positive with introducing a more electron-withdrawing substituent, following a positive linear relationship between E_1/2_ and σ, shown in Figures 3C,D (Kilgore et al., 2011a; An et al., 2012; Duan et al., 2013; Duan et al., 2015; Mognon et al., 2015; Sato et al., 2015).

Though the catalytic activity is determined by multiple factors, for the same series of catalysts with various electronic power, the introducing of electron donating groups usually tends to increase the catalytic activity of WOCs (Figures 3E,F), as predicted from the linear free energy relationships (Mognon et al., 2015; Abdel-Magied et al., 2017; Timmer et al., 2020). In their systematic study, Sun *et al.* reported how electron density affects catalytic performance of Ru-bda complex **1** (An et al., 2012; Duan et al., 2013; Sato et al., 2015; Zhang and Sun, 2019b; Corbucci et al., 2019). Proceeding by a I2M pathway, an electron-withdrawing group on **1** causes destabilization of the Ru^V^ = O species, favoring the O-O bond formation. For catalysts following WNA pathway, Rodriguez et al. (2021) reported that increasing electron-withdrawing ability can facilitate the nucleophilic attack at the Ir^V^ intermediate (a rate-determining step), therefore enhance the catalytic activity of [Cp*Ir (Xpic)NO_3_], as indicated by the correlations between σ and the measured TOF_max_.

Yoshida et al. (2010) and Abdel-Magied et al. (2017) reported that for single-site Ru complex, a more electron-donating substituent affords a smaller oxidation potential of Ru center and enhances its catalytic activity. Besides the O_2_ evolution mechanism, in their case, the influence of substituents on deactivation pathway is important in changing catalytic efficiency. In another example, a HER catalyst [NiP_2_^Ph^N_2_^C6H4X^]_2_ with electron-withdrawing -Br substituent shows a higher catalytic activity (TOF = 740 s^−1^) than its stronger competitor -CF_3_ (TOF = 95 s^−1^) because the reduced species can’t be protonated by the most electron-withdrawing groups in this family (Kilgore et al., 2011a).

Electron-donating/withdrawing substituents can also impact the UV-vis spectra. It is proposed that increasing the electronic power of substituents can improve the ligand field, hence affect the UV-vis absorptions. Take [Ru (bda) (py-4-R)] (complex **1a**) as an example, the metal-to-ligand charge-transfer (^1^MLCT) band can be largely shifted to longer wavelength when a more electron-withdrawing substituent is modified on ligand (Sato et al., 2015). In fact, a linear relationship between the energy of the lowest LMCT band of complexes [Fe (bbpen-R)]ClO_4_ (complex **11** in Figure 4) and the Hammett parameter σ was found by Lanznaster et al. (2006). The properties of complexes with different electronic effect have been summarized in Table 1.

**TABLE 1:** Properties of metal complexes with different electron-donating/-withdrawing substituents. σ Data from ref (Hansch et al., 1991).

Catalyst	Substituent	σ	E_1/2_ (V)	E_onset_ (V)	WS conditions	TON	TOF (s^−1^)	ref
**1a** R_5_ = H	N(Me)_2_	−0.83	—	—	0.365 M CAN	790	14	Duan et al. (2013)
	OMe	−0.27	−0.09^a^	—	0.365 M CAN	760	25	Duan et al. (2013)
	Me	−0.17	−0.06^a^	0.97	0.365 M CAN	2,070	33.4	Sato et al. (2015)
	H	0	−0.03^a^	—	0.365 M CAN	580	25	Duan et al. (2013)
	Br	0.23	0.01^a^	—	0.365 M CAN	4,500	115	Duan et al. (2013)
	CO_2_Me	0.45	0.05^a^	—	0.365 M CAN	—	114	Sato et al. (2015)
	CO_2_Et	0.45	—	—	0.365 M CAN	4,800	119	Duan et al. (2013)
	CF_3_	0.54	0.08^a^	1.01	0.365 M CAN	3,397	111	Sato et al. (2015)
	OMe	−0.27	0.42, 0.8^d^	1.3	0.2 M CAN	148	—	Yoshida et al. (2010)
**2**	Me	−0.17	0.48, 0.85^d^	1.37	0.2 M CAN	173	—	
	H	0	0.55, 0.9^d^	1.4	0.2 M CAN	251	—	
	OMe	−0.27	0.58, 0.93^d^	1.23	0.2 M CAN	123	—	Yoshida et al. (2010)
**3**	Me	−0.17	0.63, 0.98^d^	1.27	0.2 M CAN	184	—	
	H	0	0.68, 1.15^d^	1.29	0.2 M CAN	253	—	
**4**	OH	−0.37	—	−1.23^a^	HClO_4_, CH_3_CN	262	—	Kilgore et al. (2011a)
	OMe	−0.27	−0.88, −1.07^c^	−0.9^a^	[(DMF)H]^+^OTf^−^, H_2_O, CH_3_CN	30.5	310	
	Me	−0.17	−0.84, −1.05^c^	—	[(DMF)H]^+^OTf^−^, H_2_O, CH_3_CN	—	590	
	H	0	−0.83, −1.02^c^	—	[(DMF)H]^+^OTf^−^, H_2_O, CH_3_CN	—	590	
	PO(OEt)_2_	0.06	−0.84, −1.02^c^	—	[(DMF)H]^+^OTf^−^, H_2_O, CH_3_CN	—	500	
	Br	0.23	−0.79, −0.97^c^	—	[(DMF)H]^+^OTf^−^, H_2_O, CH_3_CN	—	740	
	CF_3_	0.54	−0.74, −0.89^c^	—	[(DMF)H]^+^OTf^−^, H_2_O, CH_3_CN	—	95	
**5a** R_5_ = H	NH_2_	−0.66	—	—	20 mM NaIO_4_	489	0.433	Rodriguez et al. (2021)
	OMe	−0.27	—	—	20 mM NaIO_4_	525	1.88	
	Me	−0.17	—	—	20 mM NaIO_4_	395	2.83	
	CF_3_	0.54	—	—	20 mM NaIO_4_	439	2.783	
**5b** R_4_ = H	NH_2_	−0.16	—	—	20 mM NaIO_4_	413	1.55	
	NO_2_	0.71	—	—	20 mM NaIO_4_	452	1.833	
**6**	OMe	0.12	—	1.3^b^	0.1 M PBS, [Ru (bpy)_3_](PF_6_)_3_	26	0.50	Abdel-Magied et al. (2017)
	Me	−0.07	—	1.28^b^	0.1 M PBS, [Ru (bpy)_3_](PF_6_)_3_	21	0.45	
	CF_3_	0.43	—	—	0.1 M PBS, [Ru (bpy)_3_](PF_6_)_3_	15	0.34	
	NO_2_	0.71	—	1.32^b^	0.1 M PBS, [Ru (bpy)_3_](PF_6_)_3_	20	0.25	
**1b** R_4_ = H	NH_2_	−0.16	0.705, 1.125^b^	—	0.365 M CAN	—	5.2	Timmer et al. (2021)
	NMe_2_	−0.15	0.685, 1.235^b^	—	0.365 M CAN	—	10.6	
	Me	−0.07	0.66, 1.15^b^	—	0.365 M CAN	—	86.9	
	OMe	0.12	0.69, 1.185^b^	—	0.365 M CAN	—	45	
	CHO	0.35	0.745, 1.155^b^	—	0.365 M CAN	—	67.7	
	Br	0.39	0.745, 1.16^b^	—	0.365 M CAN	—	330.7	
**7**	H	0	0.8^d^	—	—	390		Tseng et al. (2008)
	Me	−0.17	0.76^d^	—	—	190	
	OMe	−0.27	0.7^d^	—	—	110		
	NO_2_	0.71	1.03^d^	—	—	260		
	COOEt	0.45	0.92^d^	—	—	570		
**8**	OMe	0.12	−1.14^c^	—	[(DMF)H]^+^OTf^−^, H_2_O, CH_3_CN	—	22,000	Stewart et al. (2013)
	Me	−0.07	−1.13^c^	—	[(DMF)H]^+^OTf^−^, H_2_O, CH_3_CN	—	96,000	
	H	0	−1.12^c^	—	[(DMF)H]^+^OTf^−^, H_2_O, CH_3_CN	—	106,000	
	Br	0.39	−1.08^c^	—	[(DMF)H]^+^OTf^−^, H_2_O, CH_3_CN	—	17,000	
	Cl	0.37	−1.08^c^	—	[(DMF)H]^+^OTf^−^, H_2_O, CH_3_CN	—	15,000	
	CF_3_	0.43	−1.05^c^	—	[(DMF)H]^+^OTf^−^, H_2_O, CH_3_CN	—	4,100	
**9**	H	0	0.78, 1.28^a^	—	0.36 M CAN	16,200	0.0390	Li and Bernhard, (2017)
	Cl	0.37	0.92, 1.41^a^	—	0.36 M CAN	15,860	0.0324	
	F	0.34	0.64, 0.86^a^	—	0.36 M CAN	13,210	0.0169	
	CH_3_	−0.17	0.67, 1.15^a^	—	0.36 M CAN	14,700	0.0213	

a Potential versus ferrocene.

b Potential versus NHE.

c Potential versus Cp_2_Fe^+^/Cp_2_Fe couple.

d Potential versus SCE.

### 2.2 Intermolecular interactions

Intermolecular interactions are ubiquitous and often being used in pre-organizing molecular structures (Wang et al., 2019) or constructing dye-catalyst model for photocatalysis (Wang et al., 2022). In fact, non-covalent interactions in inter-catalyst coupling, such as hydrophobic effects, π—π stacking, halogen aromatic interactions, electrostatic interactions, off-set interaction etc., have shown impacts on the properties and catalytic activities of metal complexes in many studies (Zhang and Sun, 2019b).

#### 2.2.1 Hydrophobic effect

**FIGURE 5:**
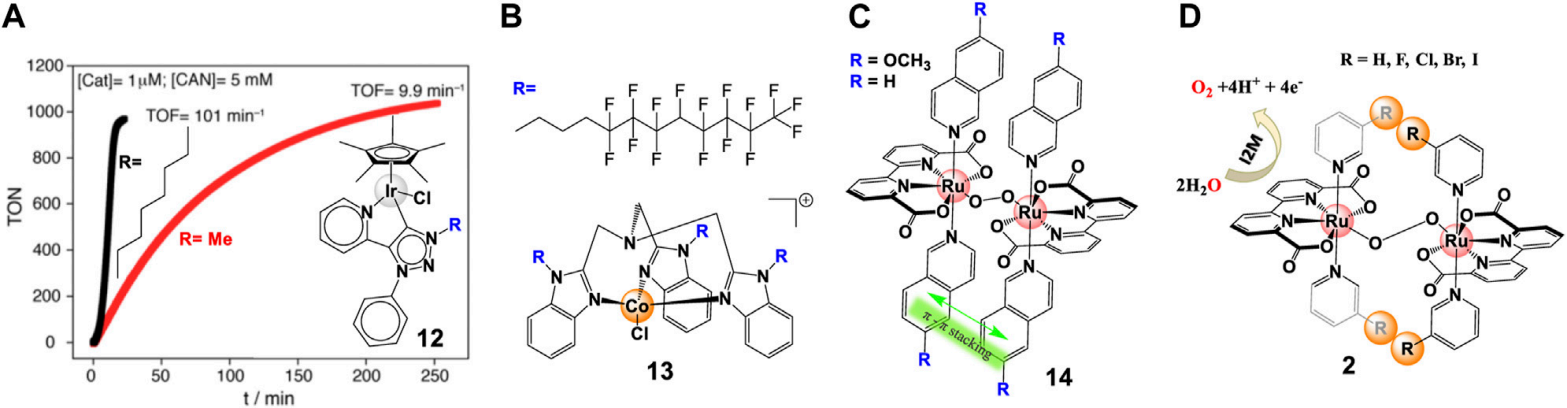
**(A)** TON of complex **12** at pH 1 measured by UV−vis spectroscopy, adapt with permission from ACS Catal. 2015, 5, 5, 2,714–2,718. Copyright 2015 American Chemical Society. **(B)** Chemical structure of complex **13**. **(C,D)** Schematic diagram for noncovalent interactions between the axial ligands for complex **14** and complex **2**.

The hydrophobic effect is often introduced by using lipophilic substituents. Generally, the hydrophobicity modification of ligands can pre-organize complexes and boost the association of two metal centers, thus improving water splitting catalytic activity. Complex **1a**, complex **12**, and complex **13** WO catalysts are good examples of this phenomenon. The hydrophobic modification on ligands improves the WO catalytic activity of Ru-bda from 22 s^−1^ to 146 s^−1^ (Liu et al., 2021). The octyl substituent in the carbene ligand of triazolylidene Cp*Ir-complexes induces the association of the iridium species, leading to a ∼10-fold increase of TOF (101 min^−1^) comparing with their methyl counterparts (TOF = 9.9 min^−1^), shown in Figure 5A (Corbucci et al., 2015). By exploiting the extreme hydrophobicity of semi-fluorinated side chains (Figure 5B), Chen et al. (2016) reported an enhanced efficient WOC, Co-(BimC_3_F_8_), with a TOF of 1.83 s^−1^, a 15-fold increase, at neutral pH without soluble cobalt, salts.

#### 2.2.2 π—π interactions

π—π interactions were extensively used in multi-components photocatalytic system, especially in promoting the electron transfer between photosensitizers and catalysts (Wang et al., 2022). Neel et al. (2017) reviewed how to exploit non-covalent π interactions for catalyst design. In chemical-driven water splitting process, π—π stacking is employed to facilitate the intermolecular interactions and accelerate bimolecular coupling. For example, complex **14** with isoquinoline (isoq) displays an order of magnitude higher TOF of 303 s^−1^ than that of 32 s^−1^ for complex **1a** with picoline (pic), as shown in Figure 5C (Duan et al., 2012b). A faster catalysis was observed by introducing MeO-isoq, which causes a more favorable π—π stacking effects in water (Richmond et al., 2014). Correlated *ab* initio calculations demonstrated that modulation of π—π stacking dispersion interactions can lower activation barrier, therefore a smaller driving force for the catalysis is obtained (Johansson et al., 2021).

#### 2.2.3 Halogen interactions

Attempts to improve the performance of WSCs also include the introduction of halogen substituents. Xie et al. (2018) studied in detail the influence of halogen substituents on the performance of complex **1b** (Figure 5D). A 10-fold enhancement of TOF (330 s^−1^) was found for R = I compare to R = H, which revealed that iodine can accelerate the O-O bond formation by facilitating the intermolecular interactions i.e. bimolecular coupling, due to its easy-polarization.

#### 2.2.4 Electrostatic interactions

**FIGURE 6:**
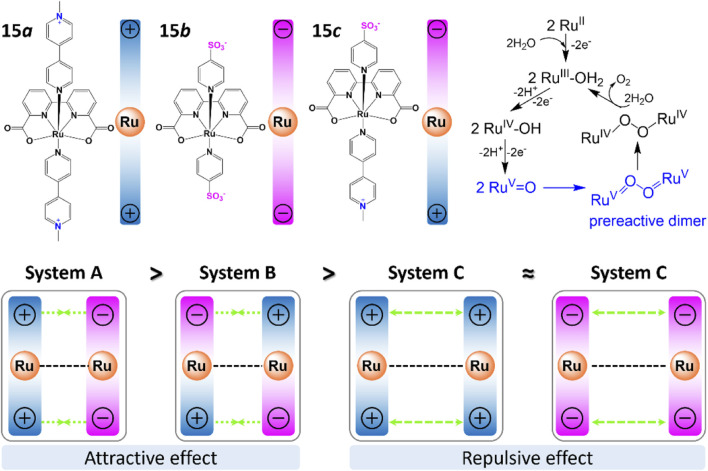
Effect of electrostatic interactions on Ru-bda catalysts, adapt with permission from J. Am. Chem. Soc. 2021, 143, 6, 2,484–2,490. Copyright 2021 American Chemical Society.

Electrostatic interactions were found to be another effective strategy to regulate binuclear catalysis. While attractive electrostatic interactions can facilitate inter-catalyst coupling and promote the catalytic performance, repulsive electrostatic interactions have a negative effect on the catalytic activity (Sampson et al., 2014; Pye and Mankad, 2017). In their recent study, Yi et al. (2021) prepared a family of Ru-bda catalysts (complex **15**, Figure 6), functionalized with positively charged Me-bpy^+^ (Nmethyl-4,4′-bipyridinium) and/or negatively charged p-SO_3_-py^-^ (pyridine-4-sulfonate) group, and identified the intermolecular electrostatic interactions by various methods. Complex **15c** and the mixture M ([**15a**]: [**15b**] = 1:1) present 8–20 times higher TOF than complex **15a** and **15b** with repulsive effects. It was proved that electrostatic interactions are benefit to the formation of pre-reactive dimers, which were key intermediates in improving the catalytic activities. In fact, Richmond et al. (2014) had discovered the electrostatic effect in their earlier report, but they attributed the lowered catalytic activity to the high steric hindrance between the Me-bpy^+^ ligands.

#### 2.2.5 Off-set interactions

**FIGURE 7:**
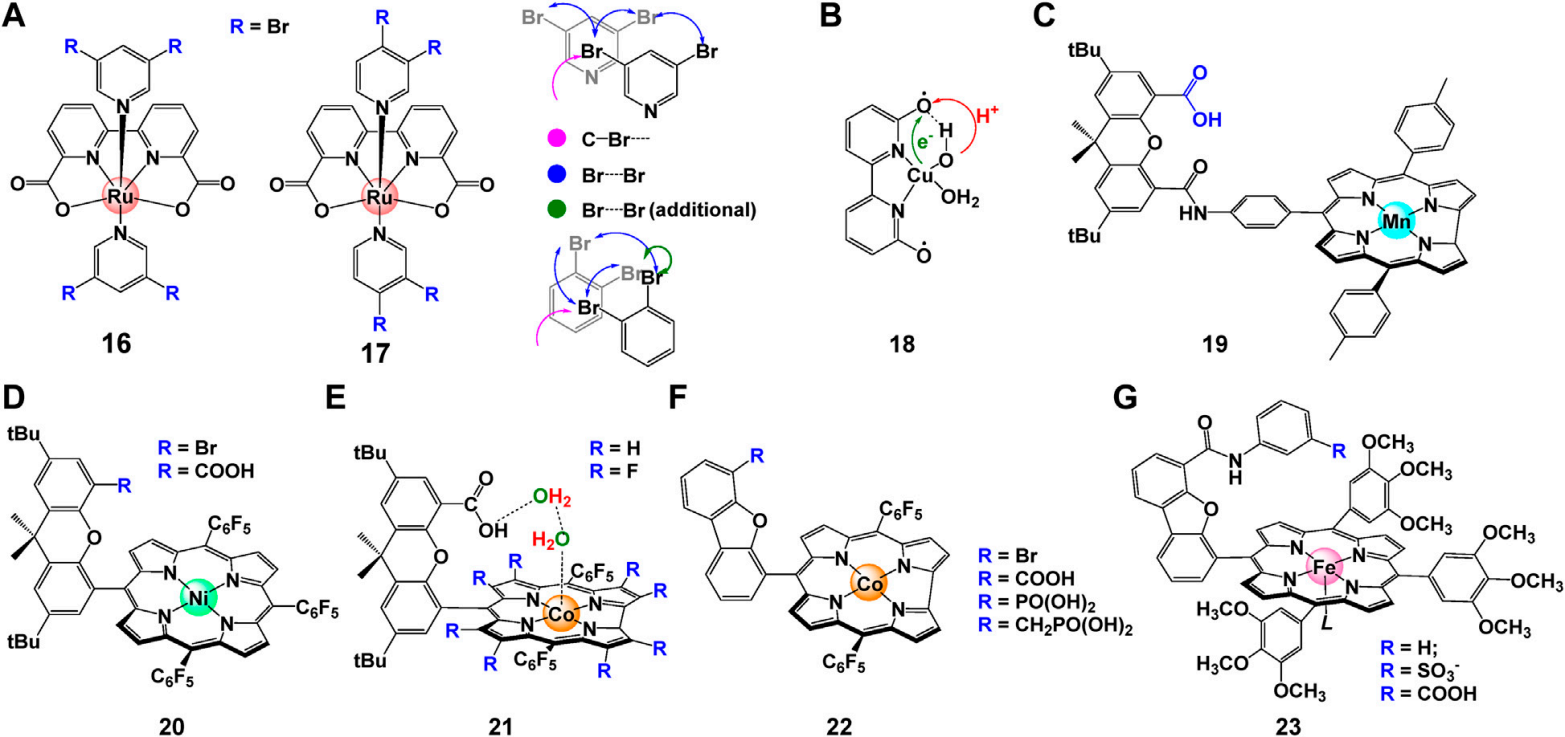
**(A)** The chemical structures of complex **16** and **17**, and the off-set interactions involved in Ru-bda catalyst, adapt with permission from Angew. Chem. Int. Ed. 2021, 60, 14504–14511. Copyright 2021 Angewandte Chemie International Edition published by Wiley-VCHGmbH. **(B–G)** Chemical structure of **(B)** copper complex **18** [Cu(bpy)(OH)_2_](Zhang et al., 2014), **(C)** Mn corrole complexes **19** (Gao et al., 2007), **(D)** nickel hangman complex **20** (Bediako et al., 2014), **(E)** cobalt hangman complex **21** (Dogutan et al., 2011; Neuman et al., 2020; Zhang et al., 2021), **(F)** cobalt corroles complex **22** (Sun et al., 2017), **(G)** iron porphyrins complex **23** (Graham and Nocera, 2014).

In addition to aforementioned intermolecular interactions, Timmer et al. (2021) discovered that off-set interactions that introduced by de-symmetrization of the axial ligands in complex **16** and **17** (Figure 7A) can provide enough space for the O-O bond formation and reduce reaction barrier. DFT calculations suggested that reduced kinetic barrier of the second-order O-O bond formation ensures high catalytic performance especially at low catalyst concentrations. For Ru-bda catalysts with isoq in the axis, the position of pyridine substituents is crucial for stacking. Instead of direct π—π interactions, the off-set interaction brought by bromide shorten the distance of Ru-Ru in the pre-reactive dimer.

#### 2.2.6 Hydrogen bonding interactions

Another attractive way to facilitate the catalytic activity is to govern proton-coupled electron transfer (PCET) process by introducing proton acceptors/donators on ligands (Young et al., 2009; Wang et al., 2019; Zhang et al., 2021). Zhang et al. (2014) proposed ligand assisted PCET process for complex **18**, where H on the 6 and 6′positions of bpy is replaced with hydroxyl groups, an internal base for proton transfer (Figure 7B). This directly lower the potential of complex about 200 mV, consequently resulting in its capability of driving WO to peroxide at a relatively low potential. Gao et al. (2007) designed bio-inspired manganese complex **19** with corrole ligands. The electrochemical data show that Mn corrole complexes **19** can catalyze oxidation of water to produce oxygen at quite low oxidation potentials, as indicated by its easy oxidation to high-valent states. Nocera *et al.* systematically studied the electrocatalytic behavior of Co/Ni hangman porphyrins complexes. They reported that owing to the pre-organization of water within the hangman cleft, the catalytic performance of these complexes (**20**, **21**) can be dramatically boosted by employing carboxyl acid as a proton acceptor (Dogutan et al., 2011; Bediako et al., 2014; Neuman et al., 2020). Later, Sun et al. (2017) synthesized complex **22** and proved that the pendant hangman carboxyl moiety can act as intramolecular base to accelerate the APT process during the O-O bond formation. In the following up study, Nocera *et al.* found that the rate of catalysis of hangman iron porphyrins complexes **23** can be affected by nearly 3 orders of magnitude by improving the hanging group’s proton-donating ability (Graham and Nocera, 2014). Recently, the impact of carboxylate unites on electrocatalyzed WO process were deeply discussed by Das et al. (2021) Same as previous studies, the free carboxylic acid/carboxylate units can provide proton donor/acceptor sites through a chemically non-innocent way, hence can improve the overpotential and activity of the WO reaction dramatically.

Clearly, these intermolecular interactions offer inspirations for future design of WSCs. The comparison between modified and parent complexes have been summarized in Table 2.

**TABLE 2:** Properties of metal complexes with different intermolecular interactions.

Complex	Factors	Substituent	E_onset (V)_	WS conditions	TON	TOF (s^−1^)	FE (%)	ref
**1**	Hydrophobic effect	CH_3_	—	1.2 mM CAN	—	22	—	Liu et al. (2021)
		CO(OC_2_H_4_)_2_(OCH_3_)	—	1.2 mM CAN	—	81	—	Liu et al. (2021)
		COOC_2_H_5_	—	100 mM CAN	—	119		Richmond et al. (2014)
		CONHC_2_H_5_	—	1.2 mM CAN	—	118	—	Liu et al. (2021)
		CONHC_4_H_9_	—	1.2 mM CAN	—	146	—	Liu et al. (2021)
**12**	Hydrophobic effect	Me	—	5 mM CAN	2,024	0.17	—	Corbucci et al. (2015)
		n-Oct	—	5 mM CAN	1,885	1.87	—	
**13**	Hydrophobic effect	C_4_H_9_	1.83^a^	MeOH, CPE^d^	—	0.12	—	Chen et al. (2016)
		CH_2_(C_2_H_5_)(C_4_H_9_)	1.81^a^	MeOH, CPE^d^	—	0.16	—	
		C_10_H_21_	1.69^a^	MeOH, CPE^d^	—	1.11	—	
		C_3_H_6_C_8_F_17_	1.61^a^	MeOH, CPE^d^	78,000	1.83	100	
**1**	π - π stacking	CH_3_	1.25^a^	0.51 M CAN	∼2,150	32	—	Duan et al. (2012b)
**14**		H	1.27^a^	0.51 M CAN	∼8,450	303	—	Duan et al. (2012b)
**14**		OCH_3_	—	0.10 M CAN	—	923	—	Richmond et al. (2014)
**2**	halogen−aromatic interaction	H	—	0.365 M CAN	580	25	—	Duan et al. (2013)
		F	1.37^a^	0.365 M CAN	—	53.8	—	Timmer et al. (2021)
		Cl	1.48^a^	0.365 M CAN	3,182	62	93	Xie et al. (2018)
		Br	1.43^a^	0.365 M CAN	4,942	101	90	Xie et al. (2018)
		I	1.36^a^	0.365 M CAN	5,280	334	96	Xie et al. (2018)
**15**	electrostatic interaction	15a	—	0.6 M CAN	—	1.54	—	Yi et al. (2021)
		15b	—	0.6 M CAN	—	1.54	—	
		15c	—	0.6 M CAN	—	12.4	—	
		[15a]: [15b] = 1:1	—	0.6 M CAN	—	34.4	—	
**1**	off-set interaction	Br	—	0.365 M CAN	4,500	115	—	Duan et al. (2013); Sato et al. (2015)
**16**		Br	—	0.365 M CAN	∼3,500	245	—	Timmer et al. (2021)
**17**		Br	—	0.365 M CAN	12,500	460	—	Timmer et al. (2021)
**20**	hydrogen bonding interaction	Br	−1.37^b^	—	—	—	—	Bediako et al. (2014)
		COOH	−1.34^b^	—	—	0.025	—	
**21**	hydrogen bonding interaction	H	0.77^b^	0.1 M PBS	—	—	—	Dogutan et al. (2011)
		F	0.87^b^	0.1M PBS	—	0.81	100	
**22**	hydrogen bonding interaction	Br	—	0.1M PBS	—	lowest	91	Sun et al. (2017)
		COOH	—	0.1M PBS	—	lower	95	
		PO(OH)_2_	—	0.1M PBS	—	higher	95	
		CH_2_PO(OH)_2_	1.27^c^	0.1M PBS	—	highest	96	
**23**	hydrogen bonding interaction	H	—	0.1 M [TEA]^+^[TsO]^−^, CH_3_CN	—	—	67	Graham and Nocera, (2014)
				0.1 M [TEA]^+^[TsO]^−^, CH_3_CN				
		SO_3_^−^	—	0.1 M [TEA]^+^[TsO]^−^, CH_3_CN	—	—	65	
		NMe_2_	—	0.1 M [TEA]^+^[TsO]^−^, CH_3_CN	—	—	65	

a Potential versus RHE.

b Potential versus ferrocene.

c Potential versus RHE.

d CPE: controlled potential electrolysis.

### 2.3 Steric hindrance

**FIGURE 8:**
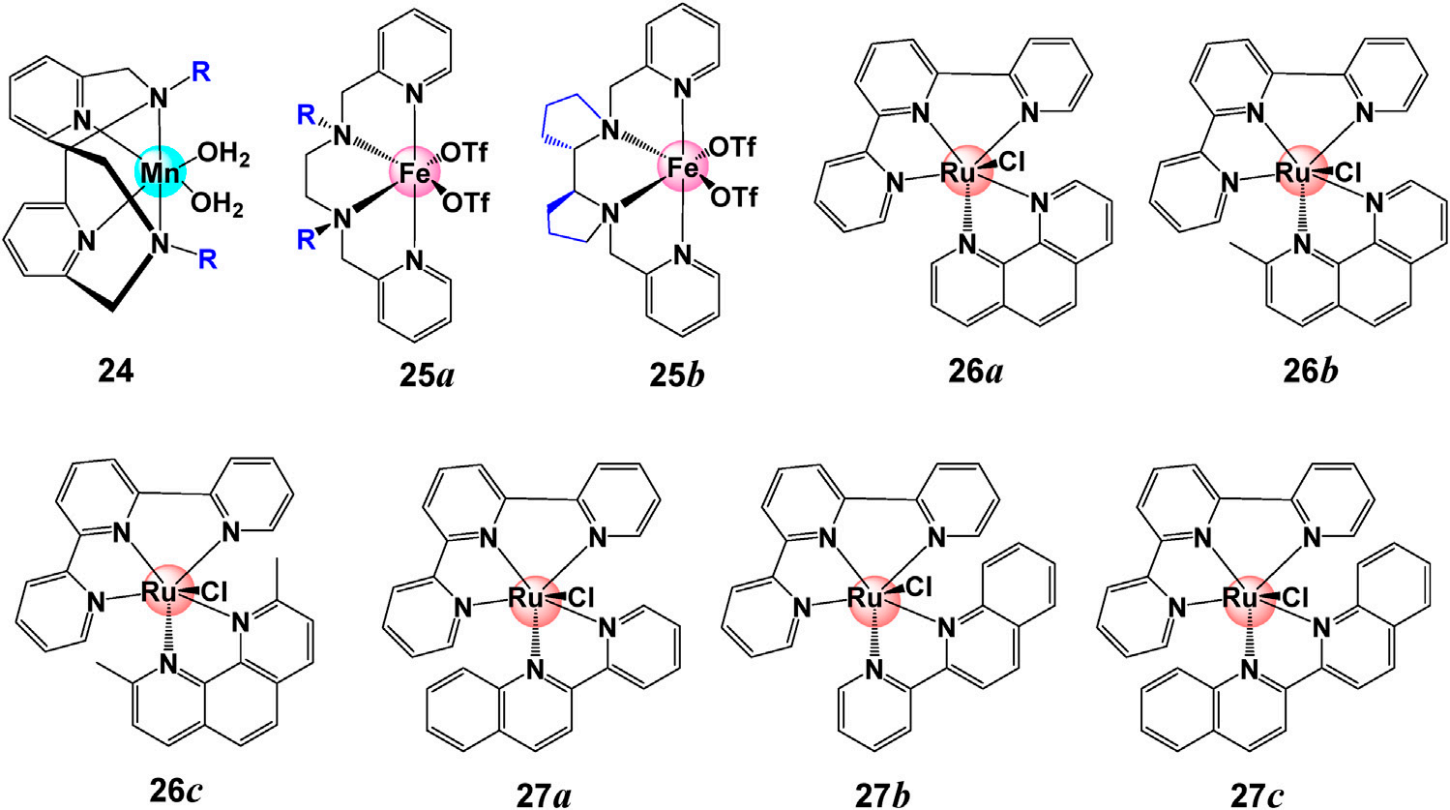
Chemical structures of complexes **24–27**.

It has been proposed that steric hindrance plays an key role in the overall catalytic rate (Richmond et al., 2019). Steric hindrance can bring changes in the properties and catalytic performance for water splitting catalysts by changing the geometry and conformation (such as bond angles and bond lengths) of metal complexes. It is interesting to note that bulky group can also switch the catalytic reactivity. Smith *et al.* reported that with small substituents (R = H, Me), complexes **24** ([(Py_2_NR_2_)Mn(H_2_O)_2_]^2+^) in Figure 8 catalytically disproportionate H_2_O_2_ in aqueous solution while this reaction is shut down with a bulkier substituent (R = tBu), but becomes active for aqueous electrocatalytic H_2_O oxidation (Lee et al., 2014; Crandell et al., 2017).

Generally, a bulky ligand can raise the activation barrier and slow down the reaction rates through steric tension in transition states or intermediates. Large substituents can shield the formation of active intermediate, hence lower the catalytic activity. Iron coordinating complex **25** with sharing a common structural topology but different geometry present different WO activity under the same condition. As indicated by L-Fe-L angle around 95–100°, the bulkier group inhibit the formation of the Fe^IV^(O)-(μ-O)Ce^IV^(OH) species (Fillol et al., 2011; Panchbhai et al., 2016). The steric hindrance is also observed for Ru-type complexes **26**. When replacing H in the phen ligand (**26a**) with one (**26b**) or two methyl groups (**26c**), the activity and stability of complex is prohibited by the methyl group. The lowest TOF of 0.005 s^−1^ and TON of 60 were observed for complex **26c** with two methyl groups, and a moderate TOF of 0.008 s^−1^ and TON of 155 were found for complex **26b** (Kaveevivitchai et al., 2012). Similar observations were also reported for complex **27**. When the benzene is proximal to Cl ligand (**27b**), the TOF is reduced as compared to complex **27a**. The activity is fully suppressed when the bpy ligand is extended by two benzene rings, as shown in complex **27c** (Kaveevivitchai et al., 2012).

**FIGURE 9:**
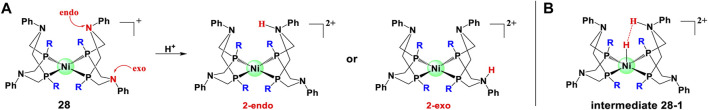
2- endo or 2-exo protonation of pendant amines in complex **28** and important intermediate for hydrogen production, adapt with permission from Inorg. Chem. 2011, 50, 21, 10908–10918. Copyright 2011 American Chemical Society.

Steric hindrance can also play its role by affecting the protonation reaction which, as we mentioned before, can influence water splitting activity. For example, for complex **28**, the protonation reaction may occur in either 2-endo or 2-exo positions as shown in Figure 9. Clearly, the 2-endo protonation site of the Ni(I) intermediate **28–1** is favored for complex **28** to enter the catalytic cycle because the strong hydride donor abilities of the metal center can accelerate the rate of H_2_ elimination from **28–1**. However, the bulky phosphine substituent can hinder the endo protonation of amines in these intermediates and also influence the hydride donor ability of [HNi (P_2_^R^NPh_2_)_2_]^+^ derivatives (Kilgore et al., 2011b; Wiese et al., 2012).

Ir-based WO complex **12** bearing pyridine triazolylidene ligands with hydrophobic octyl substituent has shown an enhanced activity as a consequence of the association of the iridium species. However, with studying the same complex with variable steric hindrance, Corbucci et al. (2019) discovered that a discontinuity of activity when change R from Me to Et, showing that steric group can inhibit the transfer of hydroperoxo or peroxo moiety from Ir intermediate to cerium, a process that slows oxygen evolution in cerium-driven WO process (Bucci et al., 2016).

**FIGURE 10:**
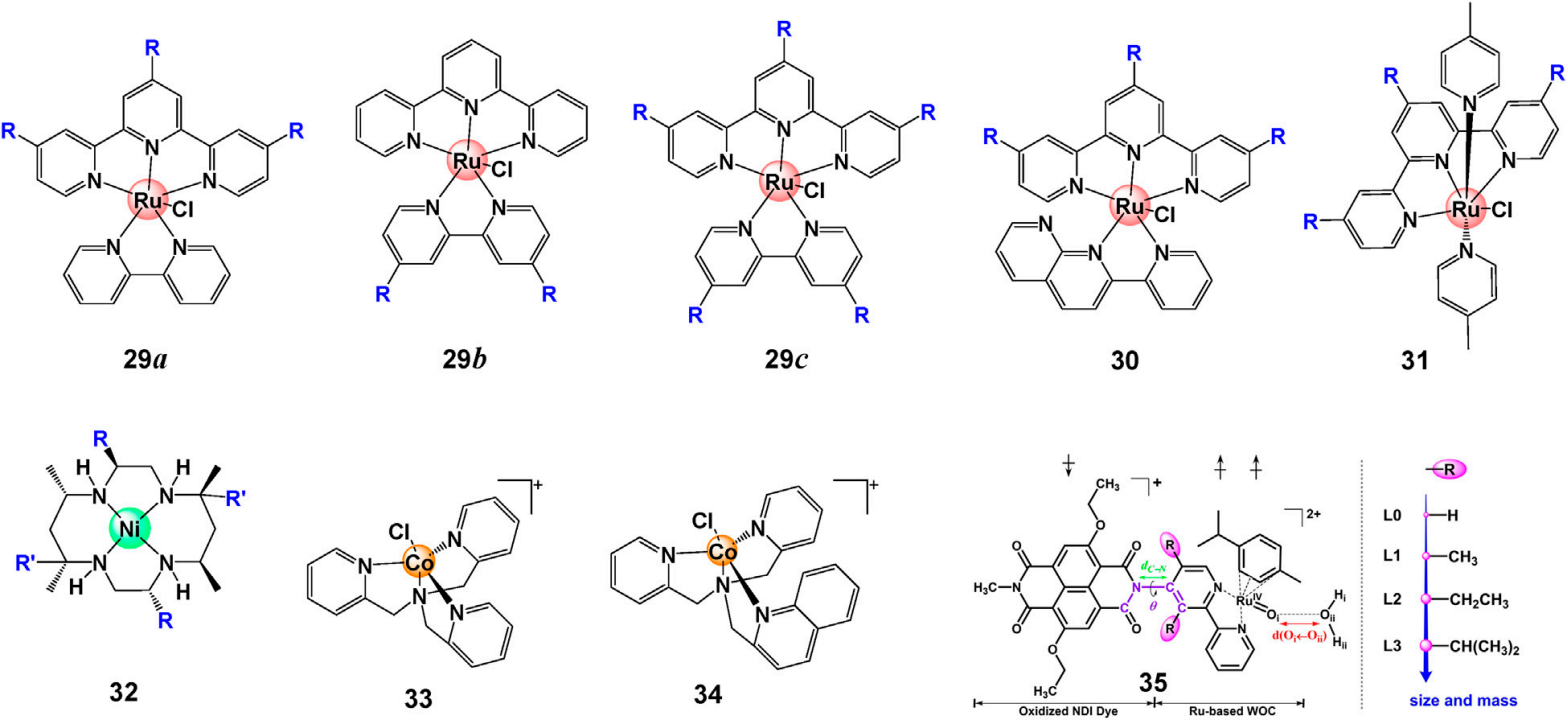
Chemical structures of complexes **29**–**34** and schematic structure of complexes **35**, adapt with permission from ChemSusChem 2021, 14, 479–486. Copyright 2020 Published by Wiley-VCHGmbH.

In contrast to the halogen interaction that improves the catalytic WO activity for [Ru (bda)(R-py)_2_], heavy halogen atoms such as iodine (I) decreases the catalytic activity of [Ru (bda)(R-isoq)_2_] due to the steric hindrance of π−π overlap, demonstrating the importance of balance between polarizability and favorable π−π interactions (Xie et al., 2018). The complicated effect of steric effect was also found on Ru(II) complexes **29**–**31**, show in Figure 10. Cyclic voltammetry and water oxidation studies illustrated that the complexes with tri-butyl-tpy ligand are easier to be oxidized because of donor attribute, hence showed boosted activity. However, these WOCs must involve a water molecule in the coordination sphere of metal to make the catalysis happen. The steric hindrance could severely inhibit the binding of water, resulting in a weakened catalytic activity when more t-butyl groups were introduced (Kaveevivitchai et al., 2012).

**FIGURE 11:**
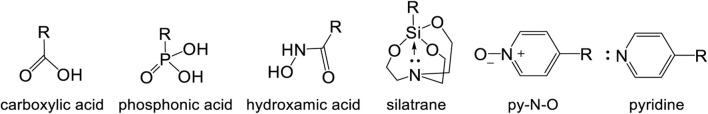
Commonly used anchoring groups for molecular water splitting process.

The steric hindrance can sometimes benefit catalysis when the isolation of transient intermediates is enabled (Chen et al., 2015). Lu *et al.* prepared three Ni complexes **32** with different number methyl group and studied their catalytic performance in aqueous buffer at pH 7.0. The catalytic activity increases with increasing the number of methyl group, suggesting that both suppressed axial coordination of phosphate anions with the Ni^III^ center and increased oxidation potentials can promote catalytic performance (Wang et al., 2017; Hessels et al., 2020).

The steric hindrance can also be built up between photosensitizers (PS) and catalysts in photocatalytic systems. In comparison with complex **33**, a dramatically diminished photocatalytic activity of **34** was observed when a quinoline is involved, indicating a steric hindrance effect (Figure 10). The steric hindrance inhibits the acceptance of electrons from PS^−^ and impedes the formation of Co(III)–H, a pivotal intermediate for H_2_ evolution, from Co(I) (Guo et al., 2021). De Groot and Buda (2021) performed constrained *ab* initio computational simulations on catalyst-dye supramolecular complex **35** and proved that efficient high-performance dye-sensitized photoelectrochemical cells can be engineered by introducing steric substituents. The properties of partial complexes with different steric hindrance effect are summarized in Table 3.

**TABLE 3:** Properties of metal complexes with different steric hindrance effect.

Complex	Substituent	E_1/2_ (V)	WS conditions	TON	TOF (s^−1^)	FE (%)	ref
**24**	H	—	3.7 M H_2_O_2_, pH 3.9	830	86	74–81	Lee et al. (2014)
	Me	—	3.7 M H_2_O_2_, pH 3.9	58,000	27	74–81	
	tBu	—	3.7 M H_2_O_2_, pH 3.9	—	—	74–81	
**25a**	Me	—	0.125 M CAN	145	0.14	—	Fillol et al. (2011)
	iPr	—	0.150 M CAN	14	0.18	—	Panchbhai et al. (2016)
**25b**	—	—	0.125 M CAN	63	0.0464	—	Fillol et al. (2011)
**26a**	H	—	0.2 M CAN	400	0.002	—	Kaveevivitchai et al. (2012)
**26b**	Me	—	0.2 M CAN	155	0.008	—	
**26c**	2^d^Me	—	0.2 M CAN	—	—	—	
**27a**	—	—	0.2 M CAN	9	50	—	Kaveevivitchai et al. (2012)
**27b**	—	—	0.2 M CAN	66	10	—	
**27c**	—	—	0.2 M CAN	—	—	—	
**28**	Me	—	[(DMF)H]OTf	10	6,700	94	(Kilgore et al., 2011b; Wiese et al., 2012)
	Benzyl	−0.83, −1.12^c^	[(DMF)H]OTf	—	130	—	
	n-Bu	−0.93, −1.23^c^	[(DMF)H]OTf	—	1,820	—	
	2-phenylethyl	−0.90, −1.16^c^	[(DMF)H]OTf	9	1,080	95	
	2,4,4-trimethylpentyl	−0.89, −1.17^c^	[(DMF)H]OTf	—	69	—	
	cyclohexyl	−0.60, −1.12^c^	[(DMF)H]OTf	—	69	—	
	phenyl	−0.84, −1.02^c^	[(DMF)H]OTf	—	720	—	
**12**	H		5 mM CAN, 0.1M HNO_3_, H_2_O	723	0.2	58	Corbucci et al. (2019)
	Me		5 mM CAN, 0.1M HNO_3_, H_2_O	1,010	0.2	81	
	Et		5 mM CAN, 0.1M HNO_3_, H_2_O	905	1.1	71	
	nPr	—	5 mM CAN, 0.1M HNO_3_, H_2_O	863	1.5	70	
	iPr	—	5 mM CAN, 0.1M HNO_3_, H_2_O	1,017	1.05	80	
	Bu	—	5 mM CAN, 0.1M HNO_3_, H_2_O	875	1.37	70	
	Oct	—	5 mM CAN, 0.1M HNO_3_, H_2_O	945	1.5	76	
**14**	F	1.38^a^	1.5 mM CAN	9,822	790	90	Xie et al. (2018)
	Cl	1.28^a^	1.5 mM CAN	26,992	364	98	
	Br	1.28^a^	1.5 mM CAN	7,371	88	67	
**29a**	H	0.80^a^	0.2 M CAN	390	20	—	Kaveevivitchai et al. (2012)
	t-butyl	0.71^a^	0.2 M CAN	667	63	—	
**29b**	t-butyl	0.80^a^	0.2 M CAN	218	33	—	
**29c**	t-butyl	0.66^a^	0.2 M CAN	94	3	—	
**30**	H	0.76^a^	0.2 M CAN	1,170	13	—	Kaveevivitchai et al. (2012)
	t-butyl	0.65^a^	0.2 M CAN	274	20	—	
**31**	H	0.75^a^	0.2 M CAN	370	50	—	Kaveevivitchai et al. (2012)
	t-butyl	0.68^a^	0.2 M CAN	310	40	—	
**32**	R = R' = H	0.77, 1.40^b^	CPE^d^	3.6	—	94	(Wang et al., 2017; Hessels et al., 2020)
	R = H, R' = Me	0.87, 1.40^b^	CPE^d^	13.0	—	97	
	R = R' = Me	1.15, 1.38^b^	CPE^d^	15.2	—	93	

a Potential versus SCE.

b Potential versus NHE.

c Potential versus ferrocene.

d CPE: controlled potential electrolysis.

### 2.4 Anchoring groups

Anchoring groups play an important role in heterogenizing molecular catalysts. By modifying anchoring groups, molecular catalysts are able to be stably loaded on semiconductors, which is beneficial to heterogenation of molecular catalysts. Zhang and Cole (2015) have conducted an extensive survey of anchoring groups used in DSSCs. Materna et al. (2017) have also thoroughly reviewed the anchoring groups for photocatalytic WO on metal oxide surfaces, including types and synthesis of surface anchors that used in DSPECs and their incorporations into molecules.

In addition to above mentioned anchoring groups as summarized by Materna et al. (2017), pyridine-N-oxide and pyridine was found to be effective anchoring groups as well (Lu et al., 2013; Mai et al., 2015). Wang et al. (2013) reported that pyridine-N-oxide can effectively bind on TiO_2_ surfaces. This guarantees the injection and adsorption of the dye molecules as indicated by an excellent IPCE of 95% and the best photon-to-electron conversion efficiency of 3.72%. Recently, (Zhu et al., 2020) found that although phosphonic acid leads to well-defined surfaces in DSPEC (dye-sensitized photoelectrosynthesis cells) assemblies, the on-surface dimerization leads to a diminished reactivity toward water oxidation compared to related monomers in solution. By contrast, the 4,4′-dipyridyl anchoring ligand of Ru-bda can maintain the monomeric structure of catalyst, affording stable photoanodes with high photocurrents and photon-to-current efficiency of 1.5% (Zhu et al., 2020).

## 3 Backbone-construction strategies of ligands

In addition to substituent’s modification, changing backbone such as from [*NN*] to [*NC*] is another effective way of enhancing catalytic performance of metal complexes. The ligands for molecular complexes can be categorized by chelating numbers: monodentate, bidentate, tridentate, tetradentate, and polydentate ligands. In most cases, one catalyst contains more than one type of ligands, but herein we focus on structures of ligands and how they affect the overall performance of WSCs. In this section, we only consider the parent ligands without extending the discussions to substituent modification strategies which have been discussed above. For simplicity consideration, we discuss tridentate, tetradentate, and polydentate ligands together in “3.3 Multidentate ligands for WSCs” section. It should be noted that all the ligands we present below are the commonly used ligands for mononuclear WSCs and they are not comprehensive lists.

### 3.1 Monodentate ligands for WSCs

A monodentate ligand has only one atom that coordinates with a metal center. Some usual atoms are nitrogen (N), carbon (C), halogen (Cl, Br, I), oxygen (O), sulphury (S) and phosphorus (P).

#### 3.1.1 [N] ligand

Pyridine, a [*N*] type monodentate ligand, is one of the earliest developed monodentate chelating ligands where a ruthenium WOC was developed and is still widely used today (Carlin, 1961; Gersten et al., 1982). It can form coordinating bond with many transition metals such as Ru (Daniel et al., 2018; Kamdar and Grotjahn, 2019), Co, (Khademi et al., 2022; Navarro et al., 2022), Ni (Wang et al., 2019), and Cu (Lee et al., 2020), etc. And be used to prepare WSCs. Pal (2018) has well explained the coordinating mechanisms and emphasized its broad application in the fifth chapter of book “Pyridine”. Recently, pyridine was noticed again by Li et al. (2021), Zhu et al. (2022) as additives retarding the back-electron transfer or electron transfer between TiO_2_ photoanode and the oxidized dye in solar water oxidation, or between an organic light absorber and a molecular WOC on a photoanode.

**FIGURE 12:**
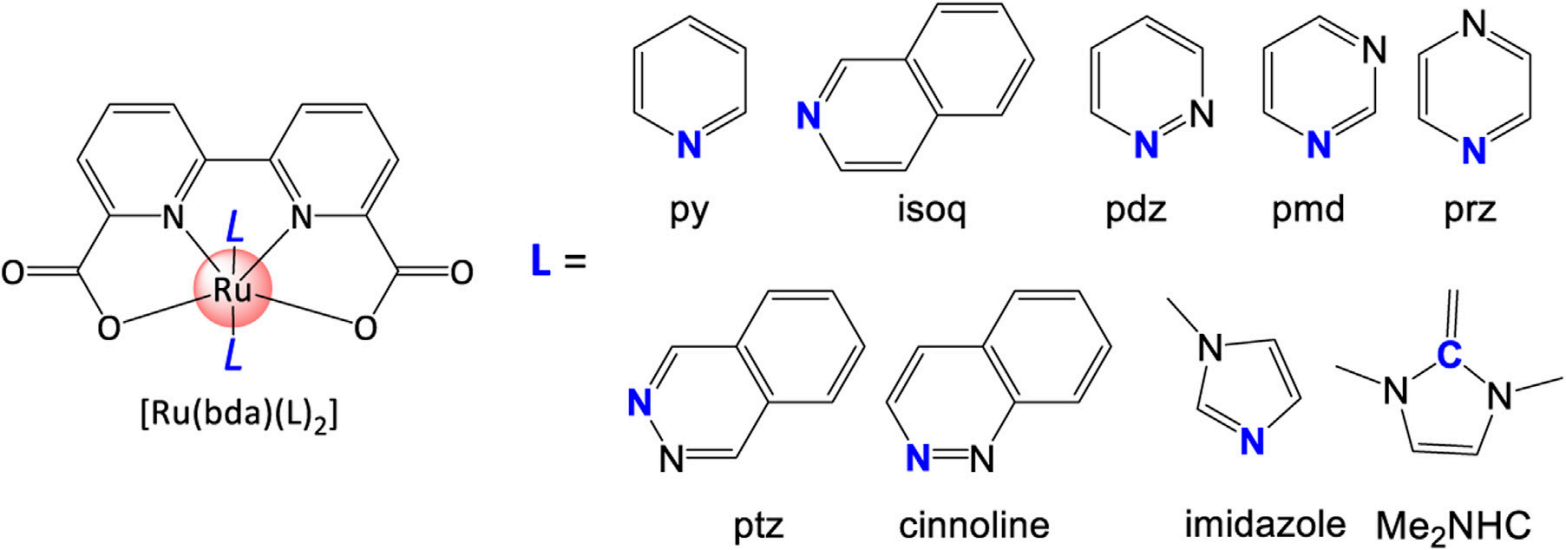
*[N]* and *[C]* ligands examples in this review.

Based on the coordinating mechanisms of pyridine, Duan et al. (2009) reported the first case of Ru-bda WO catalyst. DFT prediction implied that complex with higher HOMO energy has higher durability, i.e., stability and lifetime (Duan et al., 2012a). When change the pyridine ligand to phthalazine (pzt) ligands, an extraordinary TON of 55400 and high TOF of 286 s^−1^ were obtained. On the basis of computational analysis, a series of [*N*] ligands (Figure 12) with two benzene methine groups are occupied by “*N*” in ring, such as pyrimidine (pmd), pyrazine (prz), pyridazine (pdz), cinnoline, and phthalazine (ptz). This family of monodentate ligands shed light on the development of other types of WSCs.

Similar to pyridine-based [*N*] ligands in terms of metal-coordination properties and adjustable structures, a more electron-donating ligand, imidazole drew much attention. Imidazole has also been applied in preparing Ru-bda catalysts. The imidazole ligand can *in situ* form an active complex with the Ru^II^ center under the catalytic conditions (Wang et al., 2012). The key factor of catalytic performance in this case is that bulky ligand changes coupling between terminal oxygen atoms, and electronic properties.

#### 3.1.2 [*C*] ligand

In addition to [*N*] ligand, [*C*] ligand is another usual type of monodentate ligands such as carbene. Hetterscheid and Reek, 2011 studied [IrCp*(Me_2_NHC)(OH)_2_] (Me_2_NHC = N-dimethylimidazolin-2-ylidene) complex with a large TON of 2000. DFT calculations show that the oxidant potential of this WOC can heavily influence its catalytic water oxidation *via* various competing channels (Diaz-Morales et al., 2014; Venturini et al., 2014). Iglesias and Oro (2018) reviewed iridium–NHC catalysts, and more carbene-containing complexes will be discussed in detail in the following sections.

#### 3.1.3 Other types of ligands

**FIGURE 13:**
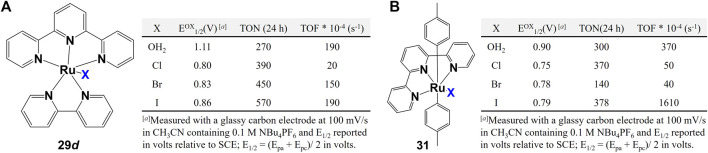
Complexes with halogen ions and aqua as monodentate ligands and their water oxidation. Data from ref (Kaveevivitchai et al., 2012).

Other monodentate ligands such as halogen ions (F, Cl, Br, I) and aqua (OH_2_) were also extensively explored. When comparing the performance of complexes with different halogen ligands, two factors are usually considered: 1) steric effect coming from the different size of halogen atoms (I > Br > Cl), 2) the O–H∙∙∙halogen hydrogen bond intensity that may affect the water/proton exchange rate (Brammer et al., 2001). Besides these two factors, however, (Kaveevivitchai et al., 2012) proposed the third possibility: halogen atoms difference may change the water splitting pathway even if the complexes have the same chemical structure but different halogen atoms. Cyclic voltammetric data of Ru catalysts **29 **d and **31** show that the aqua complexes are more difficult to oxidize than the analogous halide complexes. The WO data show that the aqua complexes performed better than the chloride and bromide complexes because the later ones require initial exchange of water for the Cl or Br ligand to produce the active intermediate species (Tseng et al., 2008; Masaoka and Sakai, 2009). However, the iodo-complexe presents unusual behavior where it catalyze considerably accelerate production of oxygen than the aqua-complex, as shown in Figures 13A,B. The unusually high initial rate for I-containing complex indicates the seven-coordinate rather than the six-coordinate intermediate pathway (Tseng et al., 2008).

### 3.2 Bidentate ligands for WSCs

In the second class of ligands, there are two coordinating atoms in each ligand. Three types of ligands are discussed according to their coordinating atoms: [*NN*] ligands, *C*-containing ligands, and *O*-containing ligands.

#### 3.2.1 [*NN*] ligand

[*NN*] ligand is the most studied type of chelating ligands and hundreds of derivatives have been developed so far. It is difficult to get an absolute conclusion of which ligand is better than the other since the catalytic performance is usually mechanism-dependent, and the same factor may have different effects but there are some general rules that can be followed, such as changing the steric geometry, electronic density around metal center, pKa, its ability of accepting/donating protons or transferring electrons, or building up PCET pathway, etc.

2,2′-bipyrimidine (bpm) and 2,2′-bipyrazine (bpz) are stronger π-acceptors and less electron-donating groups with respect to 2,2′-bipyridine (bpy), hence usually result in higher oxidation potentials but lower TON values. For example, E^o^ (Ru^III/II^) of [Ru(tpy)(bpm)(OH_2_)]^2+^ and [Ru(tpy)(bpz)(OH_2_)]^2+^ catalysts are increased relative to that of [Ru(tpy)(bpy)(OH_2_)]^2+^ by metal-ligand back-bonding to bpm or bpz in Ru(II). However, rate constants for the rate limiting step of catalysts with bpm or bpz is larger than that with bpy, demonstrating that a less energy is required for the O-O bond formation for WOCs with bpm or bpz (Concepcion et al., 2008; Concepcion et al., 2009b; Concepcion et al., 2010). Similar observations were also reported for the family of [Cp*Ir(*NN*)Cl] catalysts (**36**) where Cp* is pentamethylcyclopentadienyl and [M(*NNN*)(*NN*)(OH_2_)]^2+^ catalysts (**37**) where M is Ir, Ru, or Os (Mcdaniel et al., 2008; Concepcion et al., 2009b; Blakemore et al., 2010). DFT calculation indicates that more nitrogen atoms in ligand result in less electron density at the reactant Ru-O bond, further explicating the slightly smaller TOF of catalyst with bpy than that with bpm or bpz (Jarvis et al., 2013). Moreover, the nitrogen atoms in the chelating ligands can also change the pKa and proton-electron transfer pathway of catalytic reactions. The potential-pH diagram for bpm complex and its comparison with bpy complex revealed that PCET avoiding charge buildup leads to the thermodynamical instability of Ru(III) and leads to its poor TON performance (Concepcion et al., 2009a).

Inspired by the high performance of Mn-ligating His332 of PS II, where deprotonation process improves the catalytic activity, imidazole-containing ligands such as 2,2′-diimidazole (H_2_bim), 2-(2′-pyridyl)-imidazole (pimH), and 2-(2′-pyridyl)-benzoimidazole (pybim) were designed. An imidazole ring can not only provide more nitrogen atoms to tune electron density at metal center but also serve as a proton donor to tune proton-electron transfer pathways. In addition, the deprotonation of imidazole moiety on ligand could lower catalytic onset potential. Stott et al. (2017) studied Cu-based catalysts Cu(*NN*)(OH_2_)_2_ with introducing an imidazole ring into the bpy ligand. The experimental results showed that deprotonation of an ionizable imidazole ring can lower the metal reduction potentials and catalytic overpotentials of catalysts. Similarly, Okamura et al. (2012) modified [Ru(tpy)(bpy)(OH_2_)]^2+^ by replacing bpy with H_2_bim, a lower water oxidation potential was observed compare to analogous Ru WOCs due to the high donor power and multi-electron storage ability.

**FIGURE 14:**
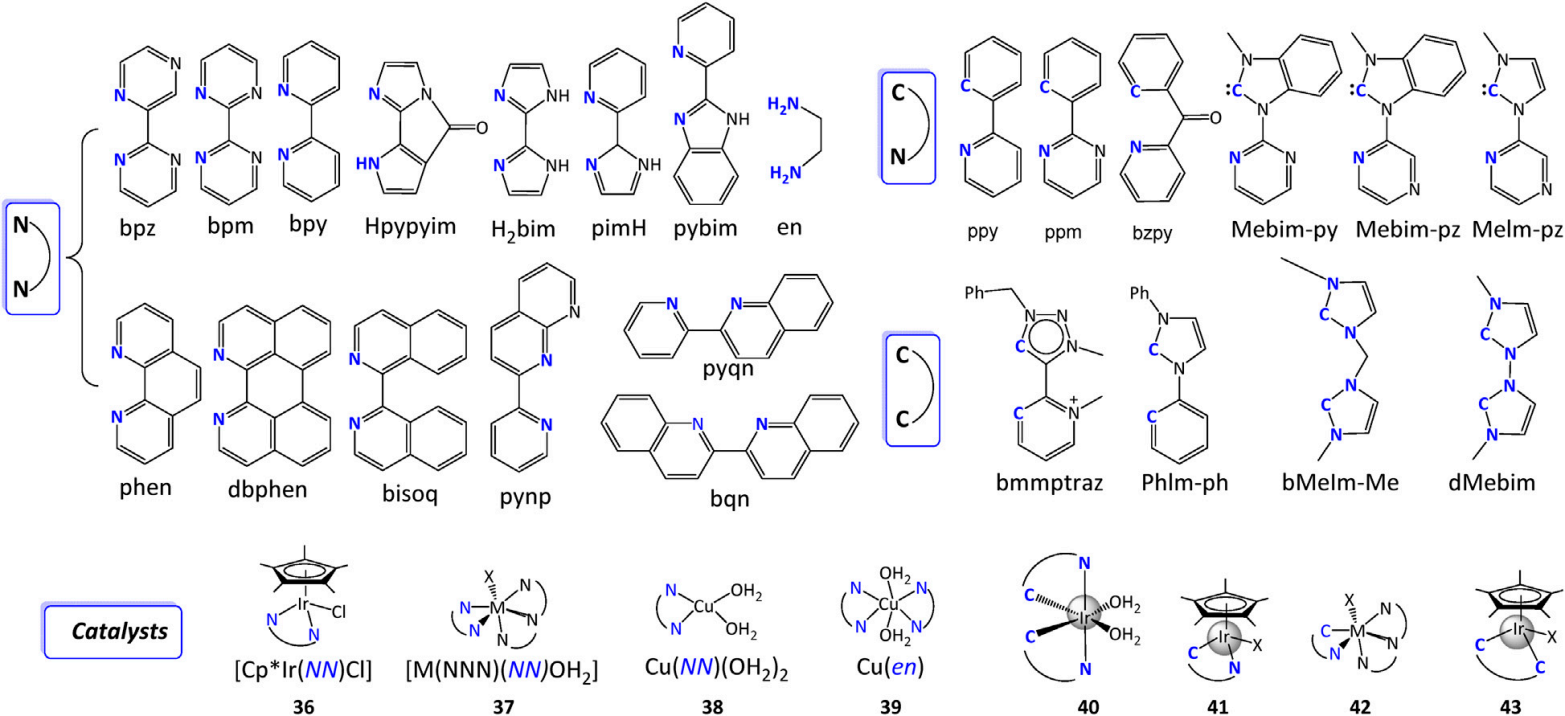
Selected [NN], [CN], and [CC] ligands and exampled water splitting catalysts discussed in this review. Coordinating atoms are labeled in blue.

In addition to changing nitrogen atoms and proton donors, steric hindrance can also influence the catalytic performance. As discussed in the preceding text, the steric impact on the activity and stability of complexes depends on water splitting conditions and mechanisms hence we can’t conclude its positive or negative effect. However, the as-needed enhanced steric effect of backbone can be generally designed either by bond fixation (to hinder rotation) such as replacing bpy with phen or by increasing the size of backbone such as modifying an extra benzene ring on ligand, as shown in Figure 14. For instance, the catalytic activity of [Ru(tpy)(*NN*)Cl]^2+^ decreased with fusing an extra ring onto the bpy ligand (pyqn) and the activity is fully suppressed when there are two phenyl rings (bqn) (Tseng et al., 2008; Zeng et al., 2015).

Besides the commonly used bipyridine type bidentate [*NN*] ligands, amine-contained ligands were also developed in recent years especially in designing earth-abundant transition-metal complexes for molecular WSCs. Amine-type ligands are advantageous as 1) their simple characterizing conditions (Lu et al., 2016), 2) the easy active conformation during OER (Lee et al., 2020) in comparison with bpy ligands. For example, by mixing a Cu(II) salt and 1,2-ethylenediamine (en), Lu et al. (2016) synthesized Cu(en) catalyst with high WO activity over a wide pH range. WO catalysis occurs in solutions from pH 7 to 10 for [Cu^II^(en)_2_(OH_2_)_2_]^2+^ complexes. At higher pH, a catalytically active layer of CuO/Cu(OH)_2_ formed on the electrode surface, producing O_2_ in a high Faradaic yield and at an overpotential superior to other Cu-based surface catalysts.

#### 3.2.2 C-containing ligand

Notably, the [*NN*] ligands generally determine the electrochemical tunability by virtue of the substituents on ligands or the intrinsic structure changes. Despite their decent catalytic activity, however, the complexity of ligands’ designs and synthesis upgrade the difficulties of obtaining homogeneous WSCs that are simultaneously simple, robust, and effective. 2-Phenylpyridine (ppy) ligand (Figure 14), where a nitrogen in bpy is replaced by carbon, is presented as an efficient ligand for WSCs owing to the formation of strong carbon-metal bond that extremely robust under typical conditions. Since it was first reported by Schmid et al. (1994) in preparing a WO catalyst [Ir (ppy)_2_(H_2_O)_2_]^+^ (**40**), ppy was proven versatile enough to generate aquo complexes with various types of cyclometalating ligands by Mcdaniel et al. (2008). In the following work, Vilella et al. (2011) reported that [Ir(O)(X)(ppy)_2_]^n^ (X = OH_2_, OH^−^ or O^2−^, depending on the pH) was the active catalytic species and X = O specie has the most basic internal base, hence demonstrated the lowest energy barrier for O–O bond formation.

**FIGURE 15:**
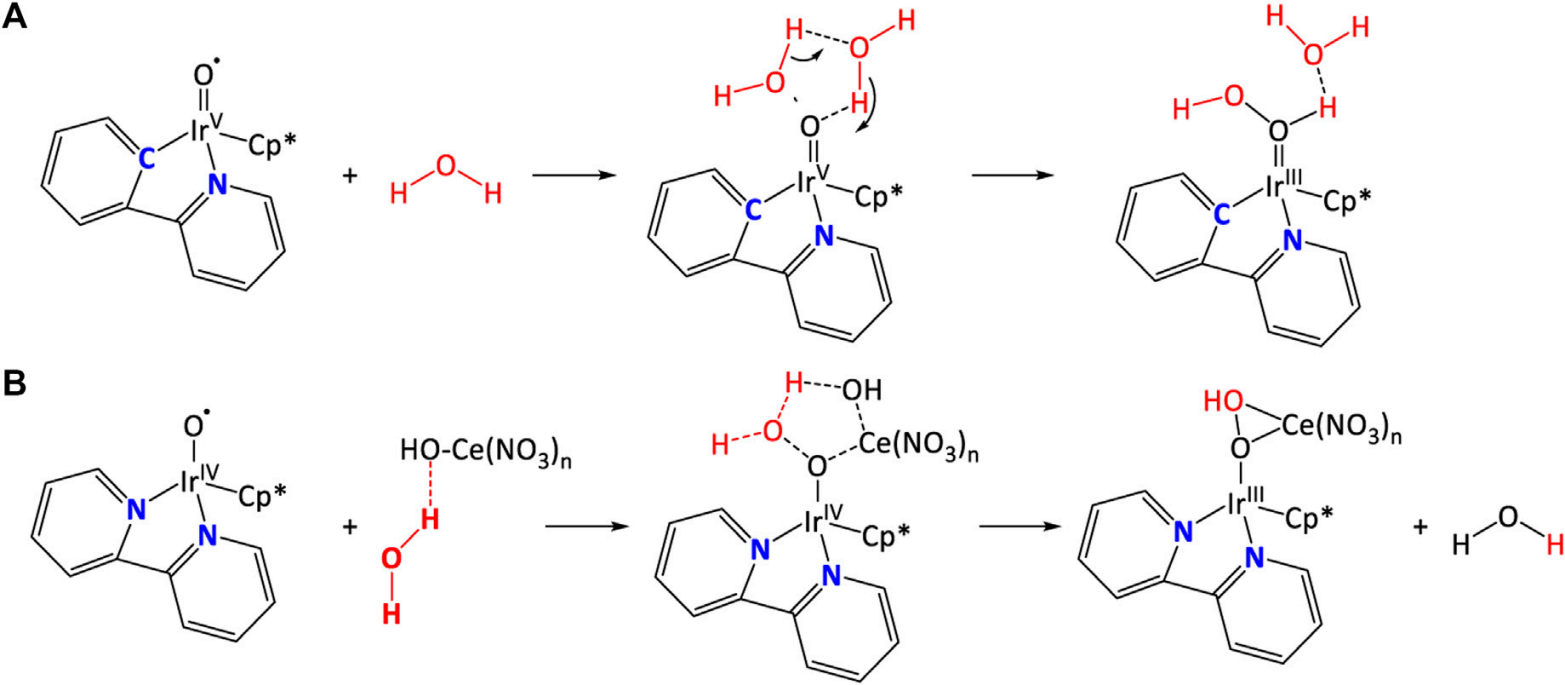
Proposed mechanism of binding water molecule for **(A)** (Cp*)Ir (ppy)Cl]^+^ (Blakemore et al., 2010), and **(B)** [(Cp*)Ir (bpy)Cl]^+^ (Bucci et al., 2016).

Crabtree *et al.* reported a series of highly active and robust cyclopentadiene (Cp*)-containing Ir complexes and compared their water splitting behaviors (Hull et al., 2009; Blakemore et al., 2010). Apparently, the robust carbon-metal bond contributes to an increase of TON for [(Cp*)Ir(ppy)Cl]^+^ (**41**), but a slight decrease of TOF in comparison with [(Cp*)Ir(bpy)Cl]^+^ (**36**), where a [*NN*] ligand was used. Subsequently, mechanism studies showed that unlike ppy-contained Cp* iridium complex, where water molecules directly interacted with the Ir^V^ = O and formed O-O bond, in the bpy analogue, [Ce^IV^(NO_3_)_3_(OH)] complex bridged the Ir^IV^-O• species and a water molecule, as shown in Figure 15 (Bucci et al., 2016). Furthermore, (Savini et al., 2010) studied a water soluble complex [(Cp*)Ir(bzpy)Cl]^+^ (bzpy = 2-benzoylpyridine) with long-term activity 2 times higher than [(Cp*)Ir(ppy)Cl]^+^ by creatively introducing a –C(O)– bridging between the two aryl rings. Obviously, the presence of an electron withdrawing group stabilizes the complex by enhancing the π-back donation from the metal. However, adding ketone group increased the flexibility of ligand and made them less robust than their ppy counterpart (Savini et al., 2011).

Another type of [*CN*] ligand is N-heterocyclic carbene (NHC)-pyridine ligands, as shown in Figure 14. Vaquer et al. (2013) synthesized aqua-Ru complexes with different number of carbene ligands and revealed a linear relationship between the number of carbene ligands and Δ E_1/2_, where Δ E_1/2_ = E_1/2_ (Ru^IV/III^) - E_1/2_ (Ru^III/II^). The enhanced Δ E_1/2_ increased the stability of Ru(III) oxidation state and therefore electrocatalytic driving force for WO. Vivancos et al. (2018) have recently reviewed the N-heterocyclic carbenes complexes in details in terms of their synthesis, catalysis, and other applications.

The strong coordination bonds between NHC ligands and transition metal centers can also significantly increase the stability of complexes containing [*CC*] ligands, such as bmmptraz. The influence of NHC on the catalytic water splitting activity was obvious. Albrecht *et al.* compared the catalytic performance of [(Cp*)Ir (bmmptraz)(MeCN)]^2+^ containing [*CC*] ligand (**43**) and its [*CN*] type counterpart (**41**) (Lalrempuia et al., 2010). Both the TON and TOF of **43** were larger than **41**. Moreover, by comparing the electrochemical behavior of [(Cp*)Ir (ppy)Cl]^+^ (**41**) and [(Cp*)Ir(PhIm-ph)Cl]^+^ (**43**), Crabtree *et al.* demonstrated that the NHC ligand on high-valent iridium has a stabilizing effect (Brewster et al., 2011).

#### 3.2.3 O-containing ligand

**FIGURE 16:**
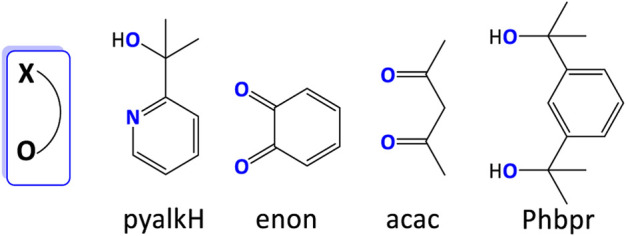
Selected O-containing ligands discussed in this review. Coordinating atoms are labeled in blue.

Some unusual bidentate ligands were also developed in the past decade. An oxidation- and dissociation-resistant [*NO*] ligand, pyalkH, has been proved useful in stabilizing unusually high oxidation states, such as Rh (Sinha et al., 2015), Ir (Shopov et al., 2015; Shopov et al., 2017), and Mn (Michaelos et al., 2016). Fisher et al. (2017) prepared Cu(pyalk)_2_ WO electrocatalyst with high activity and stability. The oxhydryl group in pyalkH provided a deprotonated site for producing alkoxide form. The complex showed a decent TOF of 0.7 s^−1^ under basic conditions (pH > 10.4). Mechanism studies suggested that only the cis form of Cu(pyalk)_2_ could convert H_2_O to O_2_. Based on the same principle, various [*OO*] ligands were reported (Figure 16) (Concepcion et al., 2010; Lippert et al., 2010), as reviewed by Limburg et al. (2012).

### 3.3 Multidentate ligands for WSCs

**FIGURE 17:**
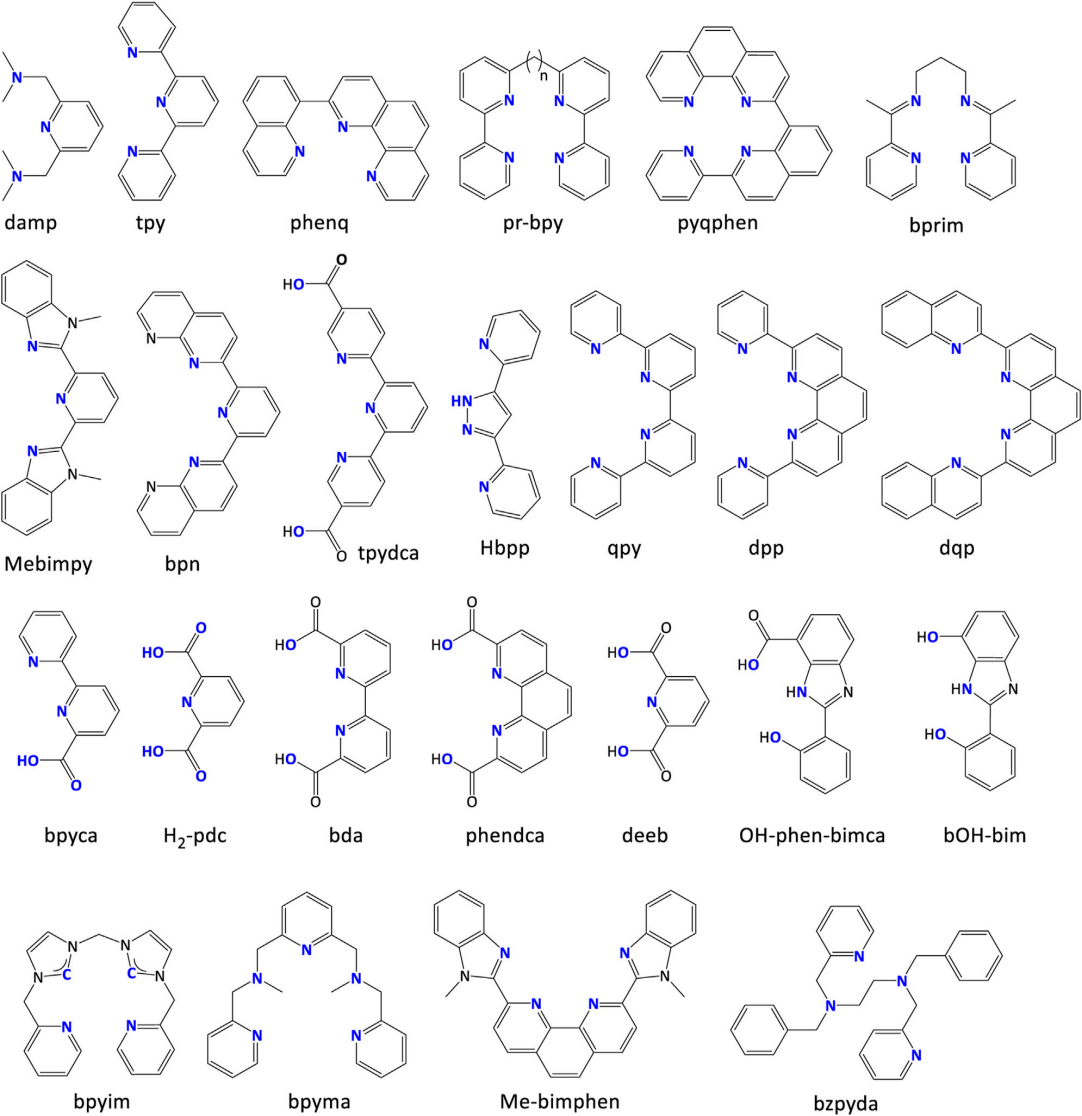
Some common multidentate ligands. Coordinating atoms are labeled in blue.

Enormous multidentate ligands for WSCs have been reported. Therefore, many reviews are available in the literature covering ligands for molecular WSCs, including some focused on WOCs (Matheu et al., 2019b; Ye et al., 2019), WRCs (Huo et al., 2019), ruthenium-based catalysts (Yu et al., 2019), nickel-based catalysts (Wang et al., 2019), etc. The designing methods for these ligands follow similar rules to bidentate ligands from the strategy point of view, including steric geometry, electronic consideration, pKa modification, etc. Considering the large number of possibilities of permutations and combinations for the coordinating atoms, here we provide a few commonplace remarks and take some common ligands as examples, shown in Figure 17.

**FIGURE 18:**
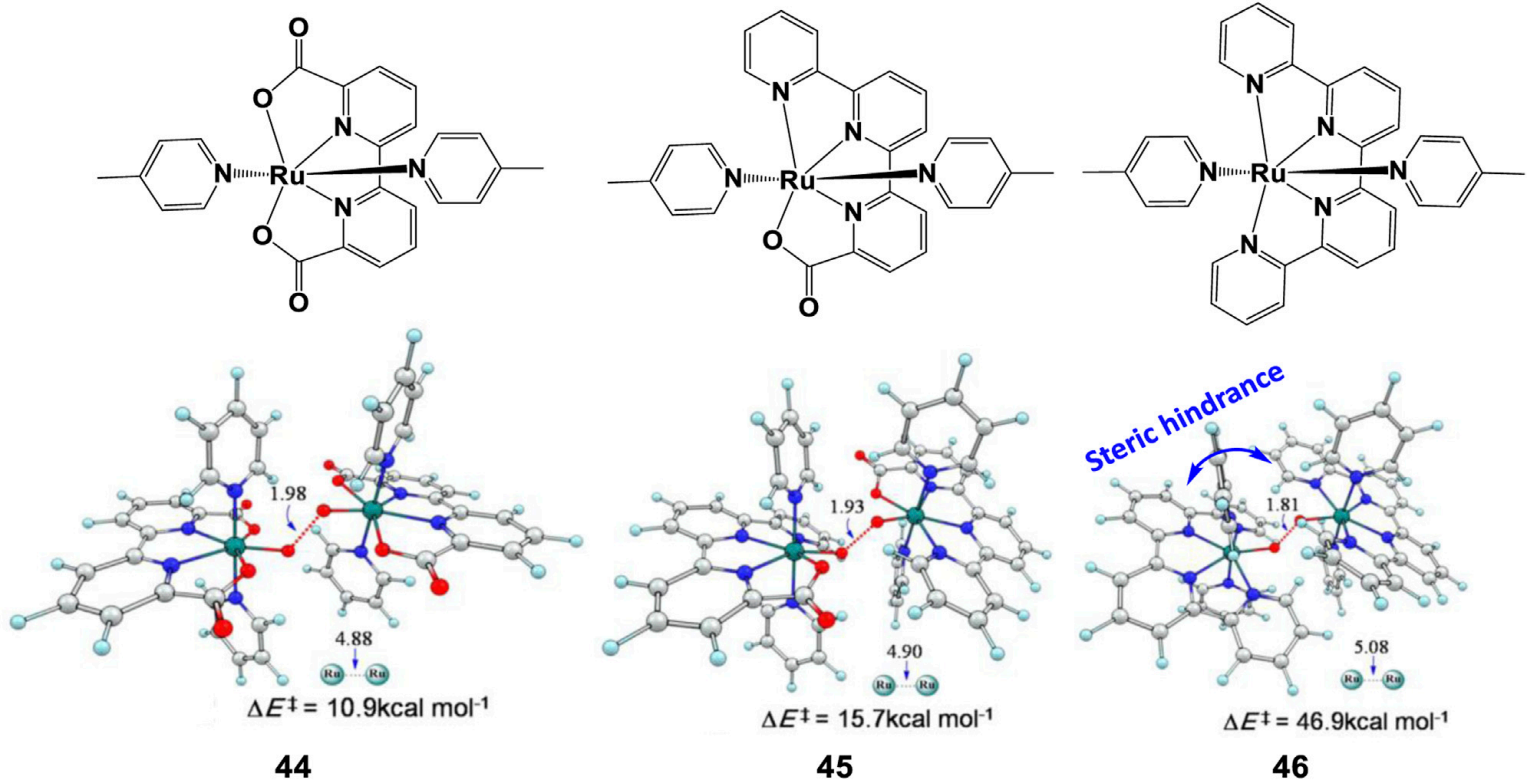
Chemical structures of complexes 44–46 and transition-state structures of O−O bond formation catalyzed with corresponding complexes following the I2M mechanism, adapt with permission from Inorg. Chem. 2014, 53, 7,130–7,136. Copyright 2014 American Chemical Society.

As discussed previously, an extended steric tension in ligand raises the activation barrier and hinder the reaction. Kang et al. (2014) compared the I2M barriers of complexes **44–46** (Figure 18), a larger increase of 31.2 kcal/mol was found from **45** to **46** than from **44** to **45** (4.8 kcal/mol). The O−O bond lengths of the three TS structures and their corresponding Ru−Ru distances prove the side effect of steric hindrance on the O−O bond formation. Therefore, for this series of complexes, a bulky ligand with large steric hindrance can severely discount the catalytic reaction rates.

**FIGURE 19:**
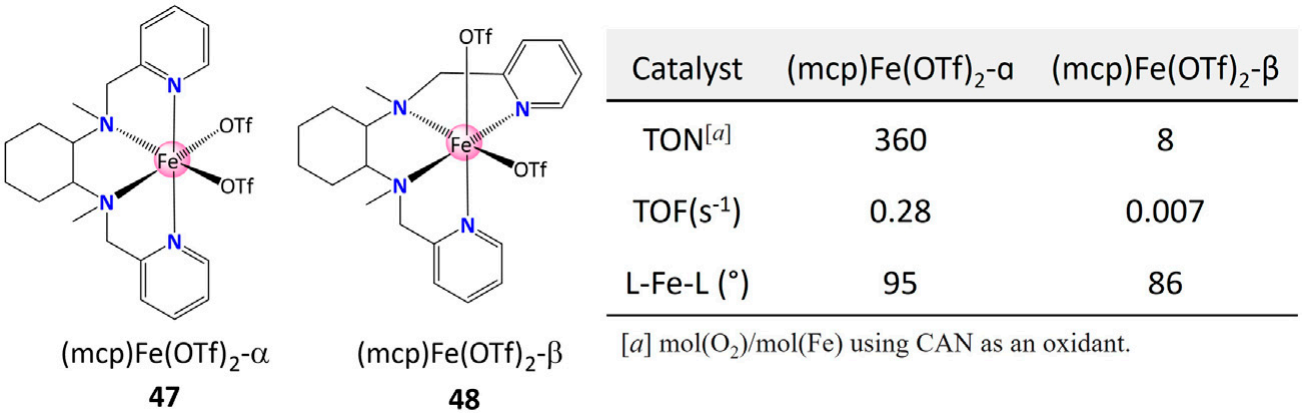
Comparison of catalytic and structural data for Fe WOCs 47 and 48. Data from ref (Panchbhai et al., 2016).

In addition to replacing coordinating site with bulky ligands, spatial configuration provides another attractive way of affecting the reactivity. Panchbhai et al. (2016) compared the catalytic (TON, TOF) and structural data for different iron-based WOCs with different geometry (**47** and **48**, Figure 19). It worth noting that the solid-state complex with smaller L–Fe–L angle (86° *versus* 95°) presented a lower TOF (0.007 *versus* 0.28) and TON (8 *versus* 360), illustrating the satirical interference on the activity.

**FIGURE 20:**
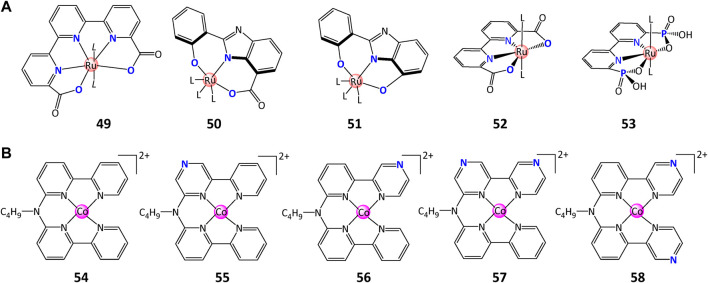
Chemical structures of **(A)** complexes containing ligand carboxylates, and **(B)** various cobalt molecular catalysts with different number and position of nitrogen in ligands.

Over the past decade, the impact of carboxylates in the ligand backbone on water splitting performance has been extensively studied and demonstrated its important role in decreasing the overpotential and increasing the WO rate. The carboxylate group(s) in ligand backbone contribute to: 1) the stability improvement of structure and photophysical properties *via* the formation of negatively charged ligands (Kärkäs et al., 2012); 2) the decreased potentials for the formation of higher valent intermediates (active species for water splitting) (Laine et al., 2015); 3) the buildup of PCET or APT pathways that can dramatically improve WO activity (Shatskiy et al., 2019; Liu et al., 2020; Das et al., 2021). In their pioneering study, Matheu et al. (2015) introduced a series of Ru-tda (tda = [2,2′:6′,2″-terpyridine]-6,6″-dicarboxylate) complex **49** with a large TOF of 8,000 s^−1^ at pH 7.0 and 50,000 s^−1^ at pH 10.0, which is 3–4 orders of magnitude better than Ru-bda at the same pH. DFT calculations manifested the key role of carboxylate as a proton acceptor in decreasing the activation energies for O–O bond formation (Matheu et al., 2015; Liu et al., 2020). This impact was also confirmed by replacing OH substituent with a -COOH group, when a 20-fold increase in TON of 4,000 was observed for complex **50** in comparison with complex **51** with TON of 180 (Kärkäs et al., 2012).

On the foundation of carboxylate ligands, Xie et al. (2016) developed a novel WO complex **53** containing phosphonate ligands which demonstrated multifunction in WO process: 1) provide effective pathways for electron donation and charge compensation; 2) increase the water solubility and stability of the complexes; 3) lower the redox-potential of high-valent metal–oxo species by charge compensation and σ-donation effects; 4) transfer protons in/out of the catalytic site to lower the activation energy for O-O bond-formation through PCET pathway. Moreover, these compounds retain the molecular activity when binding on metal-oxide surfaces, providing a desirable property for the incorporation of these catalysts in dye–catalyst assemblies (Xie et al., 2016).

Other attempts to improve the performance of WSCs were also achieved by changing the number or position of nitrogen atom in molecular cobalt catalysts, as reported by Kohler et al. (2021). They prepared five molecular Co(II) tetrapyridyl complexes **54**–**58** with different number and location of pyrazine functional groups and compared their redox potentials as well as catalytic activity. Complex **56** presented excellent activity (TOF = 3419 H_2_/Co/h, TON =1569 H_2_/Co) compared to complex **54** (TOF = 1017 H_2_/Co/h, TON =1268 H_2_/Co) while others showed inferior activity. It was explained that in the H_2_ photocatalysis process, the electron transfer from [Ru(bpy)_2_(bpy^·−^)]^+^ to Co(II) promotes activity for catalysts **56**, while for **55**, **57,** and **58**, the protons transfer promotes the overall activity. These results provided a novel option of facilitating the catalytic activity for aqueous H_2_ generation.

## 4 Conclusion

This review summarizes works on ligand designing strategies for molecular complexes aimed at achieving structure-containable molecular complexes for water splitting. From the perspective of modification position, two major strategies, substituents modification and backbone construction, are discussed. Detailed principle of how ligand modification affect catalytic performance is emphasized. The discussions are centered on electron density distribution, proton/electron-acceptance ability, bond angle, bond length, etc. Based on these consideration, various efforts including changing electron-donating/withdrawing ability, introducing intermolecular interactions, adding steric hindrance, etc. have targeted development of highly effective and stable WSCs. Comparing to the backbone construction strategy, substituents modification are more used in fine-tuning for the properties and performance of molecular catalysts.

Although some common strategies for tuning properties and performance of metal complexes are summarized in this review, as Reek Hessels et al. (2020) concluded that catalysts design rules are not universal among different transition metals and need comprehensive considerations of structure changes, mechanisms differences, and influence trends, etc. Multiple effects can be integrated in one system, and which one gains more advantages than the other needs to be analyzed case by case. In addition, the synthetic feasibility and complexity should also be considered when designing the metal complexes.
